# Increased cysteinyl-tRNA synthetase drives neuroinflammation in Alzheimer’s disease

**DOI:** 10.1186/s40035-023-00394-6

**Published:** 2024-01-08

**Authors:** Xiu-Hong Qi, Peng Chen, Yue-Ju Wang, Zhe-Ping Zhou, Xue-Chun Liu, Hui Fang, Chen-Wei Wang, Ji Liu, Rong-Yu Liu, Han-Kui Liu, Zhen-Xin Zhang, Jiang-Ning Zhou

**Affiliations:** 1 Chinese Academy of Sciences Key Laboratory of Brain Function and Diseases, Division of Life Sciences and Medicine, University of Science and Technology of China, Hefei, 230027 China; 2 Institute of Brain Science, The First Affiliated Hospital of Anhui Medical University, Hefei, 230022 China; 3 Department of Geriatrics, The First Affiliated Hospital of Soochow University, Suzhou, 215006 China; 4 Department of Neurology, Hefei Hospital Affiliated to Anhui Medical University, Hefei, 230011 China; 5 Anhui Institute of Pediatric Research, Anhui Provincial Children’s Hospital, Hefei, 230051 China; 6 School of Basic Medical Sciences, Anhui Medical University, Hefei, 230032 China; 7 National Engineering Laboratory for Brain-Inspired Intelligence Technology and Application, School of Information Science and Technology, and The First Affiliated Hospital of USTC, Division of Life Sciences and Medicine, University of Science and Technology of China, Hefei, 230027 China; 8 Department of Respiratory and Critical Care, The First Affiliated Hospital of Anhui Medical University, Hefei, 230022 China; 9 Key Laboratory of Diseases and Genomes, BGI-Genomics, BGI-Shenzhen, Shenzhen, 518000 China; 10 Department of Neurology and Clinical Epidemiology Unit, Peking Union Medical College Hospital, Chinese Academy of Medical Sciences, Beijing, 100007 China; 11 Center for Excellence in Brain Science and Intelligence Technology, Chinese Academy of Sciences, Shanghai, 200031 China

**Keywords:** Cysteinyl-tRNA synthetase, Alzheimer’s disease, Neuroinflammation, Microglia, TLR2

## Abstract

**Background:**

Microglia-mediated neuroinflammation in Alzheimer’s disease (AD) is not only a response to pathophysiological events, but also plays a causative role in neurodegeneration. Cytoplasmic cysteinyl-tRNA synthetase (CARS) is considered to be a stimulant for immune responses to diseases; however, it remains unknown whether CARS is involved in the pathogenesis of AD.

**Methods:**

Postmortem human temporal cortical tissues at different Braak stages and AD patient-derived serum samples were used to investigate the changes of CARS levels in AD by immunocytochemical staining, real-time PCR, western blotting and ELISA. After that, C57BL/6J and APP/PS1 transgenic mice and BV-2 cell line were used to explore the role of CARS protein in memory and neuroinflammation, as well as the underlying mechanisms. Finally, the associations of morphological features among CARS protein, microglia and dense-core plaques were examined by immunocytochemical staining.

**Results:**

A positive correlation was found between aging and the intensity of CARS immunoreactivity in the temporal cortex. Both protein and mRNA levels of CARS were increased in the temporal cortex of AD patients. Immunocytochemical staining revealed increased CARS immunoreactivity in neurons of the temporal cortex in AD patients. Moreover, overexpression of CARS in hippocampal neurons induced and aggravated cognitive dysfunction in C57BL/6J and APP/PS1 mice, respectively, accompanied by activation of microglia and the TLR2/MyD88 signaling pathway as well as upregulation of proinflammatory cytokines. In vitro experiments showed that CARS treatment facilitated the production of proinflammatory cytokines and the activation of the TLR2/MyD88 signaling pathway of BV-2 cells. The accumulation of CARS protein occurred within dense-core Aβ plaques accompanied by recruitment of ameboid microglia. Significant upregulation of TLR2/MyD88 proteins was also observed in the temporal cortex of AD.

**Conclusions:**

The findings suggest that the neuronal CARS drives neuroinflammation and induces memory deficits, which might be involved in the pathogenesis of AD.

**Supplementary Information:**

The online version contains supplementary material available at 10.1186/s40035-023-00394-6.

## Background

Alzheimer’s disease (AD) is the most prevalent cause of dementia in the elderly [1], characterized by a progressive decline in cognition and memory that negatively affects the lives of patients and poses a burden on individuals, families, and the society [2]. Thus, there is an urgent need for novel biological targets and drugs for AD treatment. Recently, neuroinflammation has been increasingly recognized to play a pivotal role in both the initiation and the exacerbation of AD [3, 4]. Microglial activation represents a key pathological trait of AD [5, 6]. Positron emission tomography studies have shown amyloid deposition and microglial activation in about 50% of patients with mild cognitive impairment [7]. Microglia can be persistently activated by danger-associated molecular pattern molecules, including amyloid beta (Aβ), thus resulting in an imbalance between microglial surveillance and activation [5, 8]. During AD progression, continuously activated microglia release pro-inflammatory cytokines including interleukin (IL)-1β, IL-6, tumor necrosis factor (TNF)-α, and other neurotoxic substances. These pro-inflammatory cytokines lead to aggravation of synaptic and neuronal injuries, attenuation of microglial phagocytosis, Aβ accumulation, and extracellular matrix damage [9–11]. The persistent activation of microglia creates a harmful feedback loop that triggers a chronic inflammatory state leading to neuronal damage, neurodegeneration and progressive worsening of AD [12, 13].

Aminoacyl-tRNA synthetases (ARSs) are ‘house-keeping’ enzymes that catalyze the covalent attachment of amino acids to their cognate tRNAs, which is the first step of protein translation [14]. Numerous studies have shown that altered structure and expression of ARSs are involved in various human diseases, including neurodegenerative diseases and autoimmune disorders [15]. During the past two decades, a total of 56 genetic diseases have been identified to result from variants in ARS genes [16]. Interestingly, some ARS mutations do not seem to overtly affect the enzymatic activity [17]. Throughout their evolution, ARSs progressively incorporate additional motifs and domains that are connected to neither aminoacylation nor editing [18]. ARSs are endowed with secondary biological functions related to protein–protein interactions. Prior studies have reported that some ARSs are related to the pathology of AD [19–22]. For example, tryptophanyl-tRNA synthetase has been found in extracellular plaque-like aggregates in the hippocampus of AD patients, and is inversely correlated with neurofibrillary degeneration [19, 23]. Moreover, the neurotoxicity of Aβ_25–35_ can be attenuated by resveratrol-induced autophagy through the tyrosyl-tRNA synthetase–PARP1 (auto-poly-ADP-ribosylation of the poly polymerase 1)–sirtuin 1 signaling pathway [20]. Recently, methionine-tRNA synthetase was found to be involved in the N-homocysteinylation of tau and microtubule-associated protein 1 which is associated with brain aging [21]. In addition, a previous study conducted a systematic search for global gene expression changes in the prefrontal cortex during the course of AD and showed that some ARS genes had increased expression in early Braak stages, followed by a decline in later stages [22]. However, the specific causal relationship between the expression pattern of ARS genes and AD-associated neuropathology has yet to be elucidated.

Cytoplasmic cysteinyl-tRNA synthetase (CARS) is a member of the ARS family, mainly responsible for catalyzing the ligation of cysteine to tRNA^cys^ [24]. *CARS* variants have been implicated in a multi-system, recessive disease characterized by microcephaly, developmental delay, and brittle hair and nails [25]. In addition, a novel co-segregating missense mutation in *CARS* has been identified to cause an autosomal-dominant inheritance of a Parkinsonism/Spinocerebellar-Ataxia complex in humans [26]. Moreover, the human CARS protein is embedded with UNE-C1, UNE-C2 and GST domains, of which UNE-C1 is identified as an endogenous ligand of Toll-like receptor 2 (TLR2) [27]. This structure enables CARS to be involved in immune defense beyond its primary role in aminoacylation. CARS also plays a crucial role in endogenous cysteine hydropersulfide production and is involved in the regulation of mitochondrial biogenesis and bioenergetics [28]. Previous studies have shown that CARS contributes to ferroptosis-induced cell death by inhibiting the expression of glutathione peroxidase 4 in esophageal squamous cell carcinoma [29]. Furthermore, CARS is highly expressed in most malignancies and is significantly correlated with disease progression and poorer disease prognosis [30, 31]. However, the specific role of CARS in the AD brain remains unclear.

In this study, we set out to address this question. We propose that CARS may be involved in the pathogenesis of AD through microglial inflammation. To test this hypothesis, we first analyzed the mRNA expression and protein level of CARS in the temporal cortex of AD patients and age-matched controls. Subsequently, we used mouse models to explore the role of CARS in memory impairment and neuroinflammatory processes. The underlying mechanisms of CARS-induced neuroinflammation were investigated in a cell line.

## Materials and methods

### Human brain tissue

A total of 45 postmortem human temporal cortex samples were obtained from the Netherlands Brain Bank (Amsterdam, Netherlands) and the National Health and Disease Human Brain Bank Tissue Resource Center (Zhejiang University and Anhui Medical University, China). Informed consent was obtained from the donors or their next-of-kins for use of the brain materials and medical records for research purpose. Of the donors, 35 did not have dementia or neurologic or psychiatric diseases (Additional file 1: Table S1, C1–C35, aged 16 to 93 years, 17 females and 18 males), and were included to investigate the aging effect on the expression of CARS. To assess the expression of CARS in AD, 5 control patients without dementia (Braak 0–I, Additional file 1: Table S1, C1–C5) and 10 AD patients who were further classified into mild-to-moderate (Braak III–IV, *n* = 5, Additional file 1: Table S1, AD1–AD5) and severe AD (Braak V–VI, *n* = 5, Additional file 1: Table S1, AD6–AD10) were included. The three groups were matched for age (*P* = 0.37), sex (3 females and 2 males for every group), cerebrospinal fluid pH (*P* = 0.84), and postmortem delay (*P* = 0.61). The paraffin-embedded blocks and frozen blocks were included in the tissue samples. The paraffin-embedded blocks were sectioned into 5-μm-thick slices for immunohistochemistry. The frozen blocks of the samples (Additional file 1: Table S1, C1–C5; AD1–AD10) were stored at − 80 °C until use.

### Human blood collection and serum CARS level detection

A total of 36 patients with AD (mean age 84.2 ± 1.5 years, 26 females and 10 males) and 19 control subjects (mean age 87.7 ± 1.1 years, 11 females and 8 males) (Additional file 1: Table S2) without any neurologic or psychiatric disease were recruited from the First Affiliated Hospital of Soochow University (Suzhou, China) and Hefei Hospital Affiliated to Anhui Medical University (Hefei, China). There was no difference in age (*P* = 0.13) or sex (*P* = 0.28) between the two groups. The AD patients were allocated to two groups according to disease severity, mild-to-moderate AD (*n* = 10) and severe AD (*n* = 26) [32–34]. Cognitive function was assessed by the Mini-Mental State Examination scale [35]. All subjects underwent assessment of past and present illnesses, neurologic and physical examinations and biochemical test of the blood. The study was approved by the Ethics Committees of the First Affiliated Hospital of Soochow University and Hefei Hospital Affiliated to Anhui Medical University. Informed consent was obtained from all participants or their relatives prior to their participation in the study. Blood was obtained after 12 h of fasting and coagulated for at least 30 min, followed by centrifugation at 3000×*g* for 10 min for serum isolation. Serum was decanted and stored at −80 °C until use. The serum level of CARS was assessed with the ELISA kit for CARS (OM308195, Omnimabs, Alhambra). The CARS level in the AD group was normalized to the average level in the control group.

### Immunohistochemistry and immunofluorescence staining

Immunohistochemistry and immunofluorescence staining of human brain tissues were conducted as previously described [36]. Briefly, after rehydration and antigen retrieval (Tris-EDTA, pH 9.0), 5-μm-thick sections were permeabilized with 0.3% Triton X-100 for 30 min and then blocked with 5% donkey serum in phosphate buffered saline (PBS). The slices were incubated with the following primary antibodies at 4 °C overnight: mouse anti-CARS (1:200, sc-390230, Santa Cruz, Dallas, TX), rabbit anti-CARS (1:200, NBP1-86624, Novus Biologicals, Centennial, CO), rabbit anti-NeuN (1:1000, ab177487, Abcam, Cambridge, UK), rabbit anti-Iba1 (1:500, 019-19741, Wako, Osaka, Japan), and mouse anti-Aβ (1:200, 800711, BioLegend, San Diego, CA). For immunohistochemistry, the slices were washed with PBS and incubated with biotinylated secondary goat anti-rabbit antibody (1:200, BA-1000, Vector Labs, Newark, CA) and avidin–biotin complex (Vector). The 3,3′-diaminobenzidine solution was used as a chromogen to yield brown reaction products. Photographs were obtained with a BX53 microscope (Olympus, Japan). For immunofluorescence, after washing with PBS, the slices were incubated with Alexa Fluor 488 or 594-conjugated donkey anti-mouse or anti-rabbit secondary antibody (1:200, Jackson ImmunoResearch, West Grove, PA) diluted in PBS containing 0.1% Triton X-100 at 37 °C for 1 h. Sudan black B (86015, Fluka, Buchs, Switzerland) was used to quench the endogenous autofluorescence [37]. Finally, the slices were mounted on slides and sealed with 80% glycerin for imaging. A laser-scanning confocal microscope (Zeiss LSM 880, Germany) was used to determine fluorescence.

Three slices randomly picked from each subject were used for imaging and quantification. Quantification of CARS immunohistochemistry and immunofluorescence staining was performed with 20 × and 40 × magnifications. The mean count from three to five views per slice was used for statistical evaluation. The cell counts and the intensity of CARS-immunoreactivity (ir) were analyzed with the ImageJ software (Fiji edition, NIH) in an observer-blinded manner.

### Animals

Adult male C57BL/6J (Huachuang Sino, Taizhou, China) and APP/PS1 mice (SiPeiFu, Beijing, China) at 8–10 months of age were used. The mice were group-housed, with a maximum of five mice per cage, in a colony with ad libitum access to food and water under controlled illumination and a 12 h/12 h light/dark cycle (light on from 08:00 to 20:00). The room temperature was maintained at 21–22 °C and the relative humidity was maintained at 55%. Mouse feeding and the experiments were carried out in accordance with the Guide for the Care and Use of Laboratory Animals of University of Science and Technology of China and were approved by the Animal Care and Use Committee of University of Science and Technology of China. Every effort had been made to minimize animal suffering and the number of animals used.

### Viral injection

The mice were anesthetized with sodium pentobarbital (80 mg/kg, intraperitoneal injection) and fixed in a stereotactic frame before surgery. The surgical tools and materials were sterilized with alcohol, and all procedures were applied under aseptic conditions. The mouse core body temperature was maintained at 36 °C with a heating pad. The coordinates were defined as dorsal–ventral (DV) from the brain surface, anterior–posterior (AP) from bregma and medial–lateral (ML) from the midline (in mm). To induce CARS overexpression in hippocampal neurons, recombinant adeno-associated virus (rAAV)-hSyn-CARS-P2A-EGFP (AAV2/9, 2.00 × 10^12^ viral genome (vg)/ml, Brain Case, Shenzhen, China) was injected bilaterally into the hippocampus of the male C57BL/6J or APP/PS1 mice (AP: − 2.0 mm; ML: +/− 1.2 mm; DV: − 1.80 mm). AAV-hSyn-EGFP (AAV2/9, 5.00 × 10^12^ vg/ml, Taitool, Shanghai, China) was used as the control vector. To knock down CARS in hippocampal neurons, the rAAV-U6-CMV-EGFP-SV40-pA vector expressing a short hairpin RNA (shRNA) directed at *CARS* (shCARS, AAV2/9, 6.12 × 10^12^ vg/ml, BrainVTA, Wuhan, China) or a universal scrambled shRNA (Scramble, AAV2/9, 5.12 × 10^12^ vg/ml, BrainVTA) with no homology to any transcript as a control hairpin was injected bilaterally into the hippocampus of male C57BL/6J mice. The following strands were used: shCARS sense strand 5′-CCAGAAGAUUGUGGACAAUTT-3′ and shCARS antisense strand 5′-AUUGUCCACAAUCUUCUGGTT-3′. A total of 400 nl of viral vectors was delivered into each side at a rate of 40 nl/min via a microsyringe (Hamilton, Timis Country, RO) connected to an automatic pump controller (Micro4, WPI, Sarasota, FL). After each injection, the needle was left there for 10 min and then slowly withdrawn to prevent viral leakage. After the surgery, the mice were allowed to recover for 4–8 weeks before further experiments. Mice were randomly assigned to the CARS overexpression/knockdown and control groups. Age was matched for each experiment.

### Y-maze test

Spatial recognition memory was examined by the Y-maze test as described previously [38]. The Y-maze was made of a white opaque acrylic board and consisted of three identical arms (21 cm length × 7 cm width × 15.5 cm height) placed at an angle of 120° from each other. The three arms labeled as A, B and C were randomly assigned a visual cue mounted at the distal end of an arm. To evaluate the spatial working memory, the mice were individually placed into the distal part of the arm labeled A, facing toward the center of the maze, and allowed to freely explore over an 8-min period. Arm entry was defined as all four limbs within the arm. An alternation was defined as consecutive entries into all three arms (i.e., ABC, ACB, BAC, BCA, CAB and CBA). The following formula was used to quantify the percent alternation rate: percentage alternation = spontaneous alternations/(the number of arm entries − 2) × 100%. To assess the spatial reference memory, each mouse was placed in one arm (start arm). Of the other two arms, one was closed and served as the novel arm, while the other arm was marked as the familiar arm. The mouse was allowed to explore the maze for 15 min. After 1 h, the mouse was placed back to the start arm and allowed to freely explore all three arms of the maze for another 5 min. The apparatus was cleaned with 75% alcohol and air-dried between sessions to eliminate olfactory cues. The mouse behavior in the Y maze was analyzed by the EthoVision XT 8.5 software (Noldus, Wageningen, Netherlands).

### Novel object recognition (NOR) test

The NOR test is commonly used to assess various aspects of learning and memory, particularly recognition and nonspatial hippocampal memory in rodents [39]. The NOR test was performed as described previously [40]. Briefly, the mice were individually placed in a testing square chamber, an open field box (40 cm length × 40 cm width × 40 cm height), for a 5-min habituation session and then returned to their home cage. On the second day, a training session was performed. Each mouse was placed in the box with two identical objects on the diagonal equidistant from the center and allowed to freely explore the environment for 10 min. Twenty-four hours later, a test session was performed. Each mouse was placed back to the box with one of the familiar objects replaced by a new toy (novel object), and the mouse was allowed to explore for 10 min. The time spent exploring the familiar and the novel objects were recorded. To minimize the stress of bright lighting, the experiment was conducted in a test room with dim light, with the center of the maze illuminated at approximately 20 lx. The apparatus was cleaned with 75% alcohol and air-dried between sessions to eliminate olfactory cues. Locomotion traces were recorded by a video camera and processed with the EthoVision XT 8.5 software.

### Open-field test (OFT)

OFT is often used to analyze the exploratory activity of mice [41]. The open-field apparatus was a white opaque acrylic box consisting of a 50 cm length × 50 cm width floor and four 25-cm-high walls. The floor was divided into 16 equal squares with black lines. The central area was defined as the four central squares. Each mouse was placed in one of the corners, facing the wall, and permitted to explore the open field freely for 10 min in an undisturbed environment. The apparatus was cleaned with 75% alcohol and air-dried between sessions to eliminate olfactory cues. Locomotion traces were recorded by a video camera and processed with the EthoVision XT 8.5 software.

### Elevated plus maze (EPM) test

The EPM was made up of a central zone (6 cm length × 6 cm width), two opposite open arms (30 cm length × 6 cm width) and two opposite closed arms (30 cm length × 6 cm width) with 15-cm-high walls. The EPM apparatus was raised up to 80 cm above the floor. Each mouse was placed in the central arena of the maze, facing an open arm, and left to explore for 5 min in an undisturbed environment. The apparatus was cleaned with 75% alcohol and air-dried between sessions to eliminate olfactory cues. The duration and the frequency in the open arms were recorded by a video camera and processed with the EthoVision XT 8.5 software.

### Rotarod locomotor test

To evaluate motor coordination and balance, the rotarod test was performed using a Panlab Rota Rod device (Harvard Apparatus, Holliston, MA) as described previously [42]. Briefly, the device was composed of a 30-mm-diameter rod, which was separated into five 50-mm sections by five opaque barriers. Each mouse was habituated and pre-trained on the rotating rod for three consecutive days with four trials per day prior to the formal test. In the test, each mouse was placed on the rotating rod with accelerated speed, facing away from the operator. The speed was increased from 4 to 40 rpm over 5 min. The test included three trials per day, with at least 15 min intervals. The average latency to fall was calculated from three independent trials and processed for statistical analysis.

### Mouse brain collection

Twenty-four hours after the last behavioral test, the mice were deeply anesthetized with an intraperitoneal injection of sodium pentobarbital (80 mg/kg) and sacrificed. Hippocampal tissues were collected, frozen in liquid nitrogen, stored at − 80 °C until use for western blotting. For immunohistochemistry, mice under anesthesia were transcardially perfused with cold PBS, followed by 4% (*w*/*v*) paraformaldehyde (PFA) in 0.1 M phosphate buffer (pH 7.4) for 4 min. Brains were collected and post-fixed in 4% PFA at 4 °C overnight, followed by cryoprotection via immersion in 15% (*w*/*v*) and 30% (*w*/*v*) sucrose in PBS at 4 °C for 2‒3 days until isotonic dehydration. After being frozen in the optical cutting temperature compound (TissueTek, Torrance, CA), tissues were sectioned into serial coronal slices (thickness 40 μm) through the hippocampal region with a cryostat microtome (CM1950, Leica, Heidelberger, Germany). Brain slices were stored at − 20 °C in a cold cryoprotective solution until use.

### Cloning, expression, and purification of human CARS

The human CARS (NCBI Reference Sequence: NM_01014437) full fragment was cloned and inserted into the modified PCMV vector, which contained an N-terminal Flag tag (Tsingke Biotechnology, Beijing, China), and transfected in HEK 293T cells. The cells were lysed with a radioimmunoprecipitation assay (RIPA) buffer containing a protease inhibitor cocktail (Roche, Germany) on ice. Next, the Flag-tagged CARS fusion protein was purified and eluted by anti-DYKDDDDK Affinity Beads (SA042001, Smart-Lifesciences, Changzhou, China) and 3*Flag Peptide (SLR01001, Smart-Lifesciences).

HEK 293T, SHSY5Y and BV-2 cells were maintained in high-glucose Dulbecco's modified Eagle's medium (Sigma-Aldrich, St. Louis, MO) supplemented with 10% heat-inactivated fetal bovine serum (FBS; Gibco, Grand Island, NY), 100 U/ml penicillin, 100 μg/ml streptomycin and 250 ng/ml amphotericin B (Sangon Biotech, Shanghai, China) at 37 °C in a 5% CO_2_ humidified atmosphere. We incubated BV-2 cells with Flag-tagged CARS (10 μg/ml) for 24 h to determine the effect of CARS on the TLR2/myeloid differentiation factor 88 (MyD88) signaling pathway.

### Chemotaxis assay

Chemotaxis assay was performed as previously described [43, 44]. In brief, a transwell (8.0-μm pore size culture insert, with a 6.5-mm-diameter polycarbonate membrane; 3422, Corning, Toledo, OH) system was used to assess the migration of BV-2 cells. The cell suspension was added to the upper chamber of the transwell system. Following cell adherence, Flag-tagged CARS (100 μg/ml) was added to the lower chamber, and the cells were cultured for 12 h. After that, the cells on the membrane of the transwell insert were fixed with 4% PFA, followed by staining with hematoxylin.

### Secretion test

A secretion test on SHSY5Y cells was performed as previously described [27]. In brief, after transfecting the plasmid expressing Flag-tagged CARS protein and incubation in serum-free medium for 4 h, the SHSY5Y cells were treated with different doses of TNF-α (Beyotime, Shanghai, China) for 24 h or with 10 ng/ml TNF-α for different time periods. The supernatant was centrifuged at 500×*g* for 10 min, followed by 10,000×*g* for 30 min to remove further debris. Then trichloroacetic acid (TCA) was added to the supernatant at a final concentration of 12% and incubated overnight at 4 °C for protein precipitation. The final samples were obtained by centrifugation at 1000×*g* for 15 min, followed by neutralization with 0.1 M 4-(2-hydroxyethyl)-1-piperazineethanesulfonic acid (HEPES, Sigma-Aldrich) at pH 8.0. The cells were lysed with an RIPA buffer (P0013K, Beyotime) mixed with a protease inhibitor cocktail (Roche, Basel, Switzerland) and phosphatase inhibitor (PhosSTOP; Roche) based on the manufacturer’s instructions. Finally, protein precipitation of the supernatants, as well as the cell lysate, was used for western blotting.

### RNA isolation, cDNA synthesis and quantitative real-time polymerase chain reaction (q-PCR)

Total RNA was extracted from the human brain tissue using a standard TRIzol (Invitrogen, Carlsbad, CA) method and quantified by a One Drop OD-1000 + spectrophotometer (Nanjing Wuyi Technology, China). A total of 500 ng of RNA from each sample was reverse-transcribed with PrimerScript RT Master Mix (TaKaRa, Japan). Quantitative PCR was performed using SYBR Green Master mix in a 20-μl reaction system on a StepOne platform (Applied Biosystems) for 40 cycles of 95 °C for 15 s and 60 °C for 1 min. The expression level of each target gene was analyzed using the 2^ΔΔCt^ method. Primer sequences are as follows: *CARS*, forward 5′-AGCCTCAGTCTCGAGGCCA-3′, reverse 5′-AGCTTCCATTCTGGGCCATC-3′; *TLR2*, forward 5′-GGGTTGAAGCACTGGACAAT-3′, reverse 5′- CAGAGAAGCCTGATTGGAGG-3′; *MyD88*, forward 5′-CTCCTCCACATCCCTTCC-3′, reverse 5′- CCGCAGGTTCAAGAACAGAGA-3′; *GAPDH*, forward 5′-CAAATTCCATGGCACCGTC-3′, reverse 5′-TCTCGCTCCTGGAAGATGGT-3′. *GAPDH* was used as the internal control.

### Western blotting

Total proteins were extracted from the brain tissues and cells with RIPA solution mixed with a protease inhibitor cocktail (Roche) and phosphatase inhibitor (PhosSTOP; Roche) as described above [36]. Frozen brain tissues were homogenized with a Tissuelyser (JXFSTPRP-24 L, Shanghai Jingxin, China). Total protein concentration was determined using a Bicinchoninic Acid Protein Assay Kit (Thermo Fisher Scientific). The proteins were separated on a 12% SDS polyacrylamide gel and transferred onto polyvinylidene fluoride membranes (Millipore, Bedford, MA). After 1 h of blocking with 5% nonfat milk, the membranes were incubated with primary antibody at 4 °C overnight and then processed with appropriate horseradish peroxidase (HRP)-conjugated secondary antibodies. The primary antibodies included mouse anti-CARS (1:1000, Santa Cruz, sc-390230), rabbit anti-CARS (1:1000, Novus, NBP1-86,624), mouse anti-TLR2 (1:1000, Invitrogen, 14-9021-82), rabbit anti-TLR2 (1:1000, Beyotime, AF8181), rabbit anti-MyD88 (1:1000, Absin, Shanghai, China, abs131286), rabbit anti-p-NF-κB (p-p65) (1:1000, CST, Danvers, MA, 3033), rabbit anti-NF-κB (p65) (1:2000, Abcam, ab16502), rabbit anti-p-AKT (1:1000, CST, 4060), rabbit anti-AKT (1:2000, CST, 4691), rabbit anti-p-JNK (1:1000, CST, 9251), rabbit anti-JNK (1:2000, CST, 9252), rabbit anti-p-ERK (1:1000, CST, 4370T), rabbit anti-ERK (1:2000, CST, 9102S), rabbit anti-p-P38 (1:1000, CST, 9215), rabbit anti-P38 (1:2000, CST, 8690), rabbit anti-TNF-α (1:1000, Abcam, ab183218), rabbit anti-TNF-α (1:1000, Absin, abs123966), rabbit anti-IL-6 (1:1000, Absin, abs136507), rabbit anti-IL-1β (1:1000, Absin, abs126104) and mouse anti-IL-10 (1:1000, Santa Cruz, sc-32815) antibodies. GAPDH-HRP (1:1000; KangChen, Shanghai, China, KC-5G5) was used as the internal control. All blots were detected with enhanced chemiluminescence (Thermo Fisher Scientific) on a chemiluminescence imaging system (Bioshine, Shanghai, China) and analyzed with ImageJ.

### Histology and three-dimensional (3D) reconstruction

For immunofluorescence staining of mouse brain sections, the floating sections were washed with PBS for 3 × 5 min, permeabilized with PBS containing 0.3% Triton X-100 (0.3% PBST) and blocked with 5% donkey serum in 0.3% PBST at 37 °C for 30 min. Then, the sections were incubated with primary antibodies in 0.3% PBST at 4 °C overnight. The following primary antibodies were used: mouse anti-CARS (1:200, Santa Cruz, sc-390230), rabbit anti-CARS (1:200, NBP1-86624, Novus Biologicals), mouse anti-NeuN (1:500, Millipore, MAB377), rat anti-Iba1(1:500, Abcam, ab283346), and rabbit anti-TLR2 (1:200, Beyotime, AF8181). After 30 min of room-temperature equilibrium, the sections were washed with PBS for 3 × 5 min and incubated with Alexa Fluor 594-conjugated or Alexa Fluor 405-conjugated donkey anti-rabbit, or Alexa Fluor 594-conjugated donkey anti-rat secondary antibody (1:200, Jackson ImmunoResearch) and diluted in 0.1% PBST at room temperature for 2 h. Finally, after being washed with PBS, the sections were mounted on slides and photographed with an LSM 880 (Zeiss, Germany) or FV3000 (Olympus, Japan) confocal microscope.

After immunofluorescence staining with Iba1, the sections were imaged on an FV3000 confocal microscope to obtain stereo images of the hippocampus for microglial morphology evaluation. Microglial images were obtained at 1024 × 1024-pixel resolution, and Z stacking was performed with 1.0-μm steps in the z direction. The Imaris 9.3 software (Bitplane, Switzerland) was used to quantify the process filament length, filament area, filament volume and the number of branch points, as well as to create a 3D surface rendering of the microglia [45].

### Statistical analysis

SPSS Statistics 19.0 (IBM) or GraphPad Prism 6.0 (Boston, MA) was used for statistical analysis and graphing. Filament and surface modules of the Imaris 9.3 software were used for morphological analysis of microglia in the hippocampus. Comparison between two groups was performed with two-tailed unpaired Student’s *t* test or Welch’s *t* test (for data with unequal variances). One-way analysis of variance (ANOVA) or two-way repeated-measures (RM) ANOVA was performed for comparisons among multiple groups. Correlation analysis was performed by the Pearson’s correlation test. Chi-square analysis was employed to compare categorical variables. All of the statistical analysis results are shown in the supplementary tables (Additional file 2: Table S3 and Table S4). Data are presented as the mean ± SEM. *P* < 0.05 was considered statistically significant.

## Results

### CARS expression levels in the temporal cortex increase with aging and in AD

**Fig. 1:**
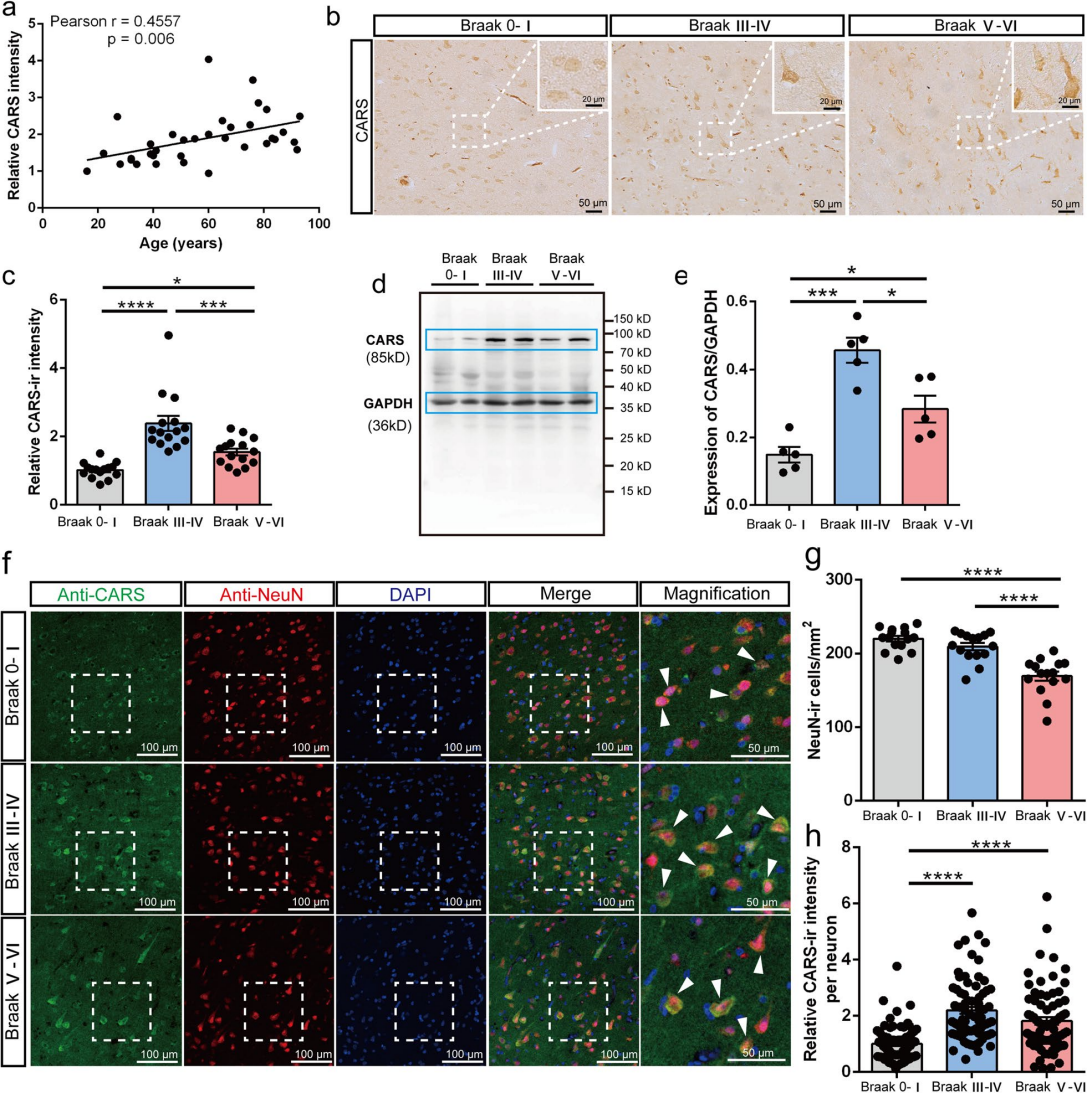
CARS protein level in the temporal cortex increases with aging and in AD. **a** Correlation between the CARS-ir intensity in the temporal cortex and age of the control subjects. **b** Representative images of CARS staining in the post-mortem temporal cortex of subjects with different Braak stages. Insets: Magnifications of the white dotted boxes. **c** Quantification of the CARS-ir intensity in panel **b**. **d** and **e** Representative images of western blotting (**d**) and quantification (**e**) of CARS protein level in the post-mortem temporal cortex of subjects with different Braak stages. **f** Representative images of neuronal CARS expression in the post-mortem temporal cortex of subjects with different Braak stages. White arrowheads denote the co-localization of CARS-ir and NeuN. **g** Quantification of the density of neurons. **h** Quantification of CARS-ir intensity per neuron in the temporal cortex of subjects with different Braak stages. Data are shown as mean ± SEM; *P < 0.05, ***P < 0.001, ****P < 0.0001

Given that aging stands as the most established risk factor for AD, we investigated the potential correlation between CARS expression in the temporal cortex and age. Immunohistochemical staining was conducted on 35 postmortem human temporal cortex samples from subjects without dementia. Interestingly, a significant positive correlation (*r* = 0.4557, *P* = 0.006) was found between the intensity of CARS-ir and age (Fig. 1a), indicating an aging-dependent increase in CARS expression. We further analyzed CARS levels in the temporal cortex of subjects at different Braak stages to determine whether its level was altered in AD. Immunohistochemistry results showed that the intensity of CARS-ir in Braak III–IV and Braak V–VI cases was higher than that in Braak 0–I controls (Fig. 1b, c). The western blotting results revealed significantly higher levels of CARS protein in Braak III–IV and Braak V–VI cases than in Braak 0–I controls (Fig. 1d, e). Interestingly, the intensity of CARS-ir and CARS protein levels in Braak III–IV cases were significantly higher than those in Braak V–VI cases (Fig. 1c, e). Furthermore, the mRNA level of CARS in Braak V–VI cases was significantly increased compared with that in Braak 0–I control subjects (Additional file 3: Fig. S1). In addition, the serum level of CARS was significantly increased in patients with mild-to-moderate AD compared with that in the control subjects (Additional file 3: Fig. S2). We next performed immunofluorescence staining on paraffin sections of the 15 samples at different Braak stages to further detect the changes of CARS expression in neurons and glial cells. In the temporal cortex of the AD subjects, CARS was co-labeled with NeuN, GFAP, and Iba1 (Fig. 1f; Additional file 3: Fig. S3, Fig. S4). To reduce endogenous autofluorescence and enhance signal-to-noise ratio in fixed human tissues, we treated the deparaffinized sections with Sudan black B after incubation with fluorescent secondary antibodies (Additional file 3: Fig. S5). As expected, the total number of neurons was significantly decreased at Braak stages V–VI (Fig. 1g). Interestingly, the average intensities of CARS-ir of individual neurons in Braak III–IV and Braak V–VI cases were significantly elevated compared with that in Braak 0–I cases (Fig. 1h). The results indicated that the expression of CARS was increased in the remaining neurons in the temporal cortex of the patients with AD.

### CARS overexpression in hippocampal neurons induces and aggravates memory impairment in C57BL/6J and APP/PS1 mice, respectively

**Fig. 2:**
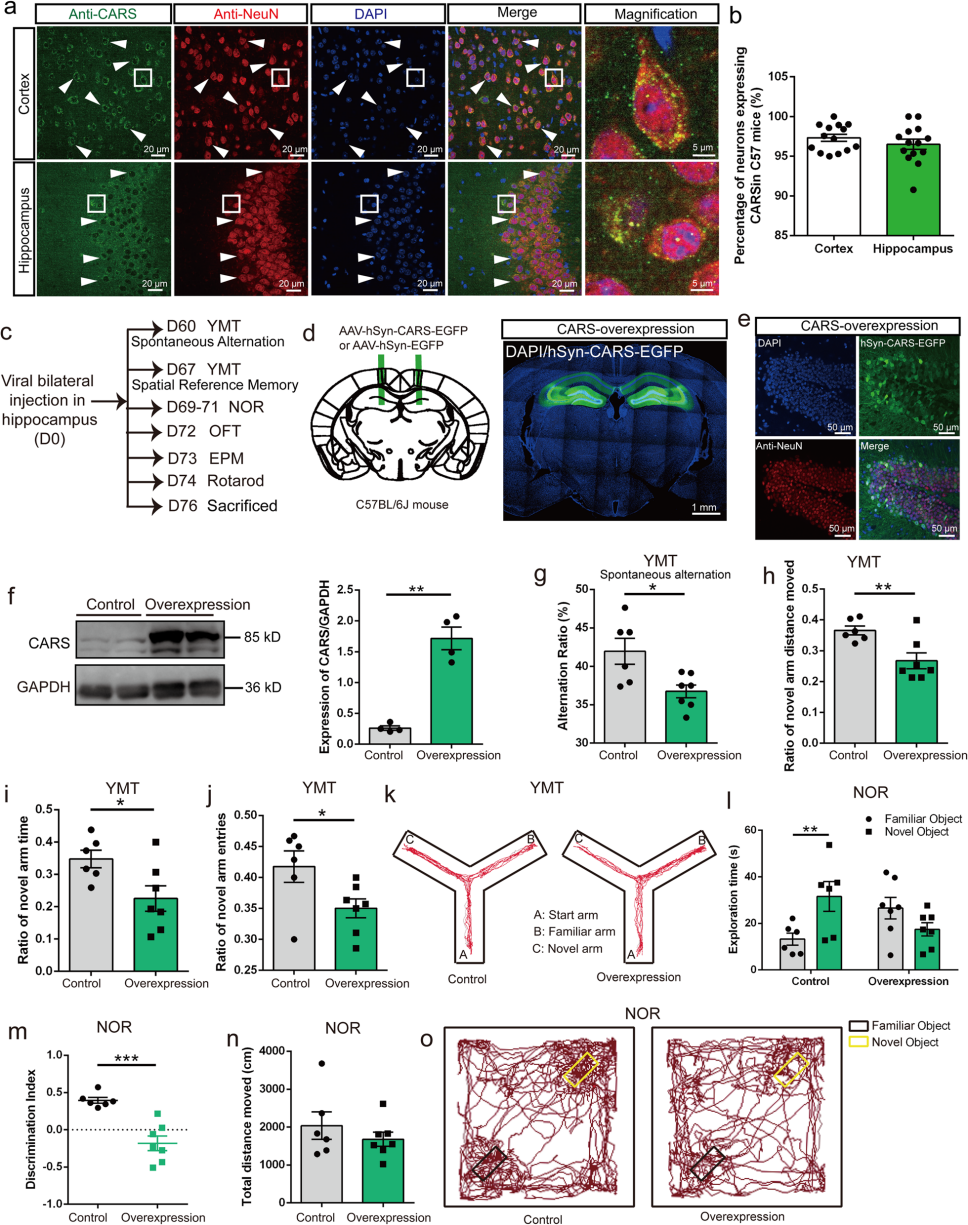
Overexpression of CARS in hippocampal neurons induces memory deficits in C57BL/6J mice. **a** Representative images showing CARS-ir neurons in the cortex and the hippocampus of C57BL/6J mice. White arrowheads denote neurons expressing CARS. **b** Percentages of neurons expressing CARS in the cortex and the hippocampus of C57BL/6J mice. **c** Schedule of viral injection and subsequent behavioral tests. **d** Left: Illustration of the injection sites. Right: Representative image of the infected hippocampus. **e** Immunostaining of NeuN in the hippocampus after the hSynapsin promoter-driven expression of CARS. **f** Left: Western blotting of CARS protein in the hippocampus of CARS-overexpressing and control mice. Right: Quantification of CARS protein level in the hippocampus. **g** Spontaneous alternation behavior in the Y-maze test (YMT). **h**–**j** Ratios of distance moved (**h**), as well as time spent (**i**) and entries (**j**) in the novel arm in the YMT. **k** Representative activity traces of the control and CARS-overexpressing mice in the YMT. **l**–**n** The exploration time on familiar and novel objects (**l**), discrimination index (**m**) and total distance moved (**n**) in the novel object recognition (NOR) test. **o** Representative activity traces of the control and the CARS-overexpressing mice in the NOR test. Data are shown as mean ± SEM; *P < 0.05, **P < 0.01, ***P < 0.001

To investigate the potential role of neuronal CARS in cognitive function, we first performed immunofluorescence staining to observe the expression of CARS in mouse neurons. The results showed that over 95% of neurons in the cortex and hippocampus expressed CARS (Fig. 2a, b). Then, we injected AAV-hSyn-CARS-P2A-EGFP into the bilateral hippocampi of C57BL/6J mice to overexpress CARS in neurons (Fig. 2c–e). As shown in Additional file 3: Fig. S6, the AAV-hSyn-CARS-P2A-EGFP (overexpression) or AAV-hSyn-EGFP (control) virus infected over 90% of the hippocampus in the C57BL/6J mice. The level of CARS protein in the hippocampus was markedly increased in the CARS-overexpressing mice compared with that in the control mice (Fig. 2f, Additional file 3: Fig. S7). Two months after injection, the two groups of C57BL/6J mice were subjected to a series of behavioral tests (Fig. 2c, g–o). In the Y‐maze test, the CARS-overexpressing group exhibited a lower rate of spontaneous alternation (Fig. 2g), as well as significant decreases in the ratio of distances moved in the novel arm, the ratio of novel arm time, and the ratio of novel arm entries (Fig. 2h–k). In the NOR test, the control mice showed a strong preference for exploring the novel object, while the CARS-overexpressing mice spent a similar amount of time exploring both objects (Fig. 2l). The CARS-overexpressing mice also showed a decreased discrimination index compared to the control mice (Fig. 2m, o). Importantly, no significant differences in the total distance moved were observed between the two groups (Fig. 2n), indicating that CARS overexpression did not affect motor capacity.

The two groups of mice were also subjected to a series of behavioral tests for anxiety and motor activity assessments, including an OFT, an EPM test and a rotarod test (Additional file 3: Fig. S8). No significant difference was detected in the total distance moved (OFT), time and entry in the central area (OFT), time and entry in the open arms (EPM), or the latency to fall (rotarod), revealing that overexpression of CARS in the hippocampal neurons of the C57BL/6J mice had no effect on the anxiety level or locomotor activity. The above results indicated that CARS overexpression in hippocampal neurons induced memory impairment in C57BL/6J mice. To further investigate the role of neuronal CARS in memory impairment in AD, we next injected AAV-hSyn-CARS-EGFP or AAV-hSyn-EGFP into the bilateral hippocampi of the APP/PS1 mice (Additional file 3: Fig. S9a–d, Fig. S10). Behavioral tests revealed significant decreases in the alternation ratio, ratio of novel arm duration and ratio of novel arm entries in the CARS-overexpressing APP/PS1 mice compared with the control APP/PS1 mice (Additional file 3: Fig. S9e–i). In addition, no significant behavioral differences were detected between the two groups in the NOR, OFT, EPM, and rotarod tests (Additional file 3: Fig. S9j–s). The results suggested that CARS overexpression in the hippocampal neurons aggravated memory deficits in APP/PS1 mice.

### Neuronal overexpression of CARS activates microglia and the TLR2/MyD88 pathway in the hippocampus

**Fig. 3:**
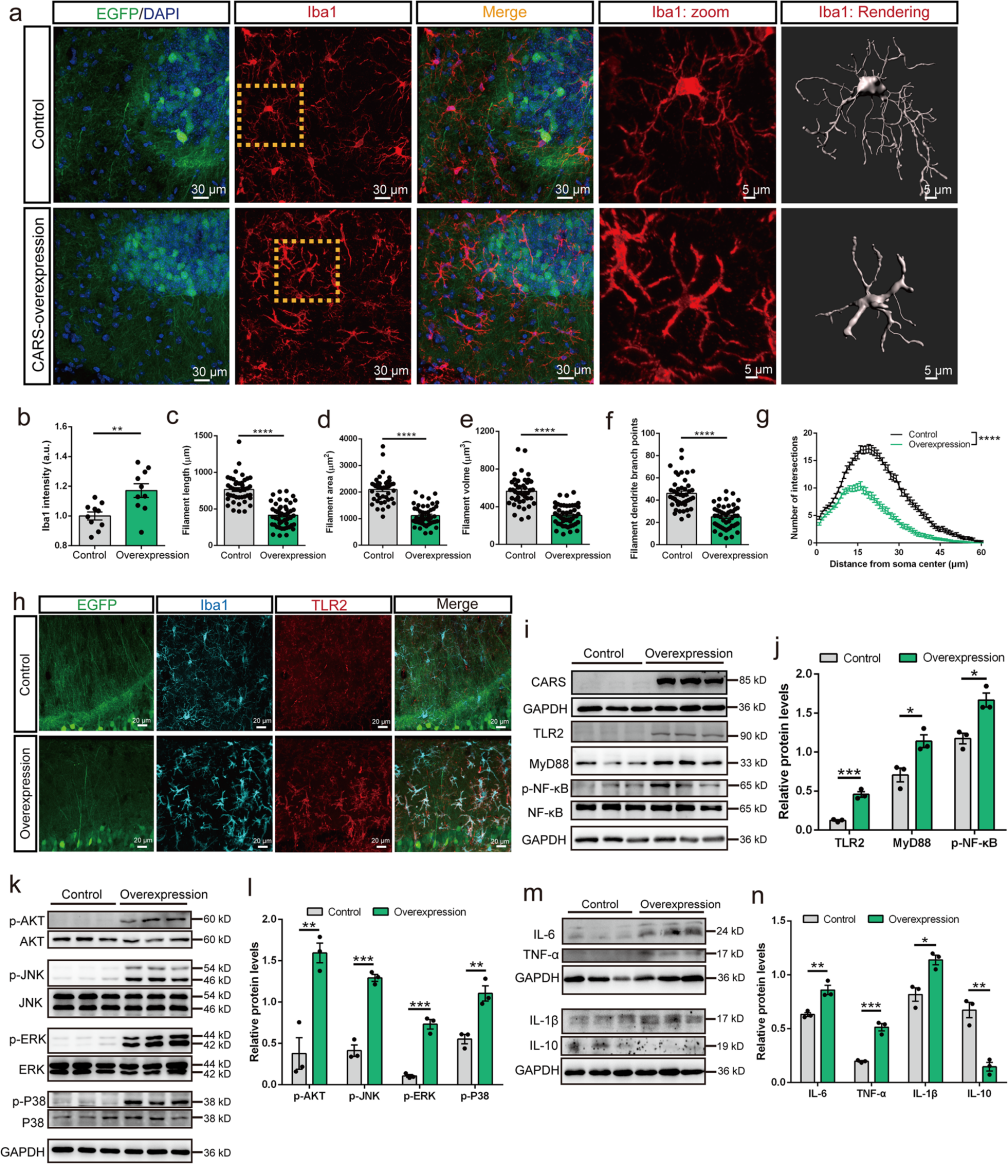
Neuronal overexpression of CARS activates microglia and the TLR2/MyD88 pathway in the hippocampus. **a** Representative images of Iba1 immunostaining (red) and 3D reconstruction (gray) of microglia in the hippocampus of the naïve C57BL/6J mice treated with AAV-hSyn-CARS-EGFP (CARS-overexpression or overexpression) or AAV-hSyn-EGFP (control). The areas indicated with dotted lines are magnified and shown in the “Iba1: zoom” images. **b** Quantification of Iba1 fluorescence intensity in the hippocampus of the control and CARS-overexpressing mice. **c**–**f** Imaris-based automated quantification of Iba1^+^ microglial filament length (**c**), filament area (**d**), filament volume (**e**), and numbers of dendrite branch points (**f**) in the hippocampus of the control and the CARS-overexpressing mice. **g** Sholl analyses of microglial morphology in the control and the CARS-overexpressing mice. **h** Representative images of immunostaining for Iba1 (blue) and TLR2 (red) in the hippocampus of the control and the CARS-overexpressing mice. **i**, **j** Representative western blotting images (**i**) and quantification (**j**) of TLR2, MyD88 and p-NF-κB protein levels in the hippocampus of the control and the CARS-overexpressing mice. **k**, **l** Representative western blotting images of p-AKT, p-JNK, p-ERK and p-P38 proteins (**k**) and quantification (**l**) of the ratio of p-AKT/AKT, p-JNK/JNK, p-ERK/ERK and p-P38/P38 in the hippocampus. **m**, **n** Representative western blotting images (**m**) and quantification (**n**) of IL-6, TNF-α, IL-1β, and IL-10 protein levels in the hippocampus. Data are shown as mean ± SEM; *P < 0.05, **P < 0.01, ****P < 0.0001

Microglia are closely related to the cognitive impairment and pathology of AD [46, 47]. To investigate the potential mechanism of CARS-induced memory impairment, we examined the effects of neuronal CARS overexpression on microglia in the hippocampus of the C57BL/6J mice. The results showed that Iba1 intensity was significantly increased in the CARS-overexpressing mice (Fig. 3a, b). We then analyzed the morphology of microglia, which is known to be closely related to their activation state. Semiautomatic quantitative morphometric measurements of microglia based on 3D reconstruction (Fig. 3a) revealed that the filament length, filament area, filament volume, and the number of branching points were all significantly decreased in the CARS-overexpressing mice compared with those in the control mice (Fig. 3a, c–f). Sholl analysis showed that with neuronal CARS overexpression, the highly ramified microglia in the hippocampus became less branched, as indicated by the less number of intersections at a distance of 10–43 μm from the cell body (Fig. 3a, g). A previous study reported that the CARS protein was embedded with the UNE-C1 domain, which is an identified endogenous ligand for TLR2 [27]. Microglia are the only type of cell in the central nervous system that expresses nearly all identified TLRs, including the constitutively expressed TLR2 [48]. The levels of TLR2, MyD88, which is the common adaptor protein involved in TLR signaling, and p-NF-κB proteins were significantly increased in the hippocampus of the CARS-overexpressing mice (Fig. 3h–j). Furthermore, the levels of phosphorylated AKT, JNK, ERK and p38 were also significantly increased (Fig. 3k, l). Importantly, neuronal CARS overexpression increased the levels of pro-inflammatory cytokines including TNF-α, IL-6 and IL-1β, a well-documented characteristic of microglial activation, and reduced IL-10 expression (Fig. 3m, n).

**Fig. 4:**
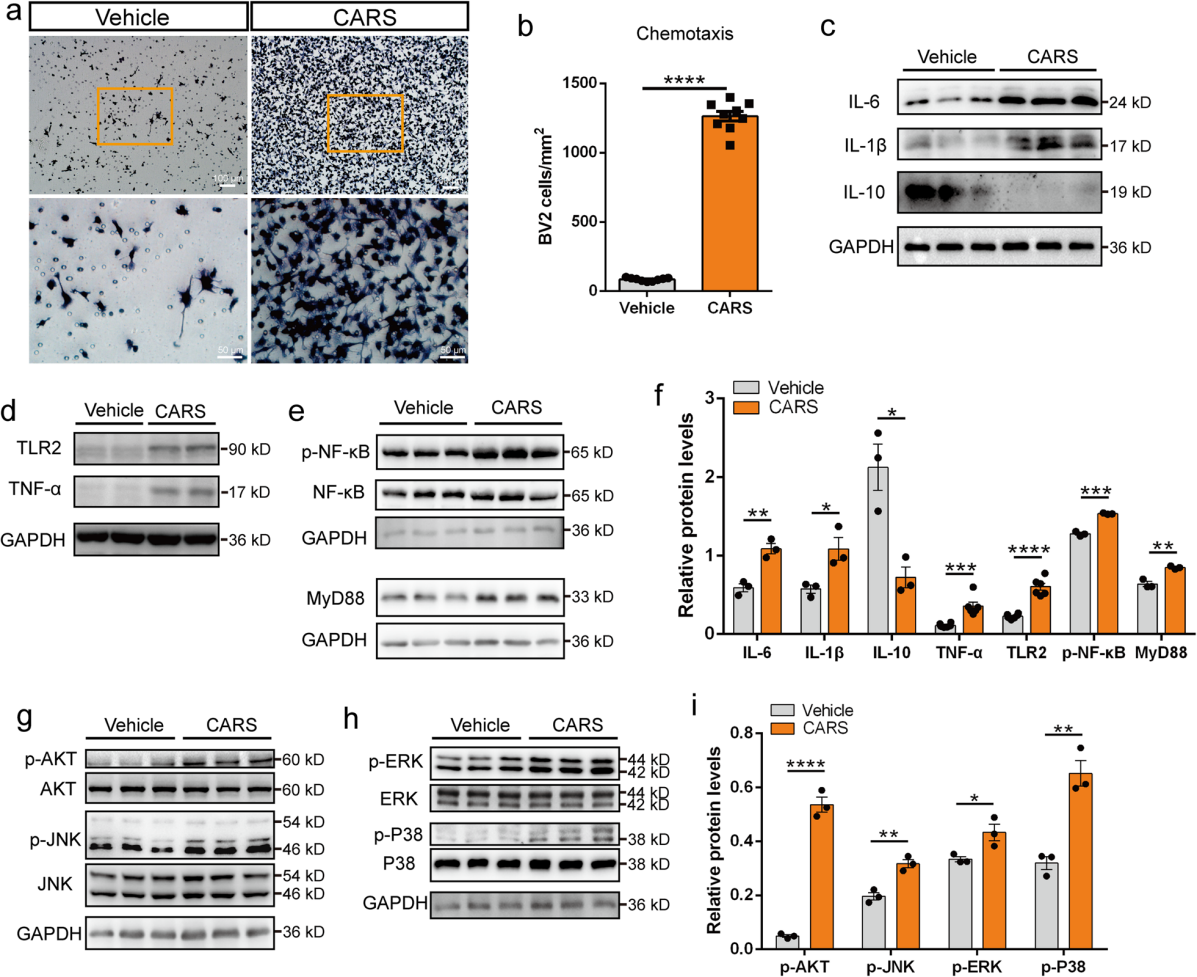
CARS activates microglia and the TLR2/MyD88 pathway in vitro. **a** Representative images of adherent BV-2 cells stained with hematoxylin in the vehicle and CARS treatment groups. BV-2 cells were treated with CARS (100 μg/ml) for 12 h. Magnifications of the yellow boxes are shown in the lower panels. **b** Quantification of BV-2 cell density in the vehicle and CARS treatment (100 μg/ml, 12 h) groups. **c**–**f** Representative western blotting images (**c**–**e**) and quantification (**f**) of IL-6, IL-1β, IL-10, TLR2, TNF-α, p-NF-κB, and MyD88 protein levels in the BV-2 cells treated with CARS (10 μg/ml) for 24 h. **g**–**i** Representative western blotting images (**g** and **h**) and quantification (**i**) of p-AKT, p-JNK, p-ERK and p-P38 protein levels in BV-2 cells treated with CARS (10 μg/ml) for 24 h. Data are shown as mean ± SEM; *P < 0.05, **P < 0.01, ***P < 0.001, ****P < 0.0001

We further investigated the effect of neuronal CARS overexpression on microglia in the APP/PS1 mice. The results showed that neuronal CARS overexpression in the hippocampus resulted in significant reductions of the filament length, filament area, filament volume and the number of branching points of microglia in APP/PS1 mice (Additional file 3: Fig. S11a–e). The Sholl analysis revealed a decreased number of intersections from the microglial cell body in the hippocampus of the CARS-overexpressing APP/PS1 mice (Additional file 3: Fig. S11f). Additionally, the western blotting results indicated significantly increased levels of TLR2, MyD88, p-ERK, and p-P38 (Additional file 3: Fig. S11g–j, Fig. S12). Interestingly, the p-JNK level was significantly decreased (Additional file 3: Fig. S11i–j). In addition, no significant differences in the p-NF-κB and p-AKT levels were observed between the two groups (Additional file 3: Fig. S11g–j). Regarding pro-inflammatory cytokines, TNF-α was significantly upregulated, whereas IL-6 and IL-1β levels were not significantly altered (Additional file 3: Fig. S11k, l). These results indicated that overexpression of CARS induced activation of microglia and the TLR2/MyD88 signaling in both C57BL/6 J and APP/PS1 mice, with differential effects on downstream signaling pathways. Interestingly, consistent with the findings in mice, we found significant increases in the protein and mRNA levels of TLR2 and MyD88 in the postmortem temporal cortex samples of human AD subjects compared with the control subjects (Additional file 3: Fig. S13a–c). Overall, the results indicated that neuronal CARS overexpression activates microglia and the TLR2/MyD88 pathway in the hippocampus.

### CARS knockdown in the hippocampus has no significant effect on memory in C57BL/6J mice

To investigate the role of neuronal CARS in cognition, we employed a viral-based shRNA delivery strategy to knock down the expression of CARS in the hippocampal neurons of the C57BL/6J mice (Additional file 3: Fig. S14a–c). The knockdown group (shCARS) showed a significant decrease of CARS in the hippocampus compared with the control group (Scramble) (Additional file 3: Fig. S14d). The decrease of neuronal CARS did not induce any significant alteration in cognitive, emotional, or motor-related behavioral performance (Additional file 3: Fig. S14e–s). The 3D reconstruction results revealed that the morphology of hippocampal microglia remained unaltered with the knockdown of neuronal CARS (Additional file 3: Fig. S15a–f). Similarly, no significant alterations in the levels of TLR2, pro-inflammatory cytokines or signaling molecules (p-JNK, p-ERK and p-P38) were observed. Interestingly, the MyD88 protein level was significantly decreased, while that of p-AKT was significantly increased in the CARS-knockdown mice (Additional file 3: Fig. S15g–l, Fig. S16). These results revealed that the neuronal CARS knockdown in the hippocampus of C57BL/6J mice had no significant effect on memory or TLR2-related microglial neuroinflammation.

### CARS activates microglia and the TLR2/MyD88 signaling pathway in vitro

The BV-2 cell line was used to elucidate the potential functional relationship between CARS and microglia. CARS protein was purified using anti-DYKDDDDK Affinity Beads (Additional file 3: Fig. S17). After treatment with CARS (100 μg/ml) for 12 h, the number of BV-2 cells migrating toward the bottom chamber was significantly increased compared with that with vehicle treatment (Fig. 4a, b). Furthermore, some classic inflammatory cytokines including IL-6 and IL-1β, as well as TNF-α, were significantly elevated, while IL-10 was significantly decreased in the whole-cell lysates of BV-2 cells at 24 h after CARS treatment (10 μg/ml) (Fig. 4c, d, f; Additional file 3: Fig. S18). We next explored the mechanism underlying the CARS-induced microglial activation. After CARS (10 μg/ml) exposure, the levels of TLR2, adaptor protein MyD88, p-NF-κB, as well as phosphorylated AKT, JNK, ERK and p38 were increased (Fig. 4e–i), suggesting that CARS treatment induces activation of the TLR2/MyD88 pathway and the NF-κB, AKT and MAPK signaling in BV-2 cells. Interestingly, in human neuroblastoma SHSY5Y cells, treatment with different doses of the pro-inflammatory factor TNF-α (1, 5, 10 ng/ml) significantly increased the level of intracellular CARS protein and induced secretion of CARS protein at 24 h (Additional file 3: Fig. S19a, b, e, f). Furthermore, after incubation with TNF-α (10 ng/ml) for 3, 6, 12, and 24 h, the protein levels of both intracellular CARS and secreted CARS in the supernatant increased over time (Additional file 3: Fig. S19c, d, g, h). The results showed that the expression and secretion of CARS were increased in a dose- and time-dependent manner after TNF-α treatment, indicating that CARS might contribute to the positive feedback loop of the inflammatory response.

### CARS accumulates within dense-core plaques along with the recruitment of ameboid microglia in the temporal cortex in AD

**Fig. 5:**
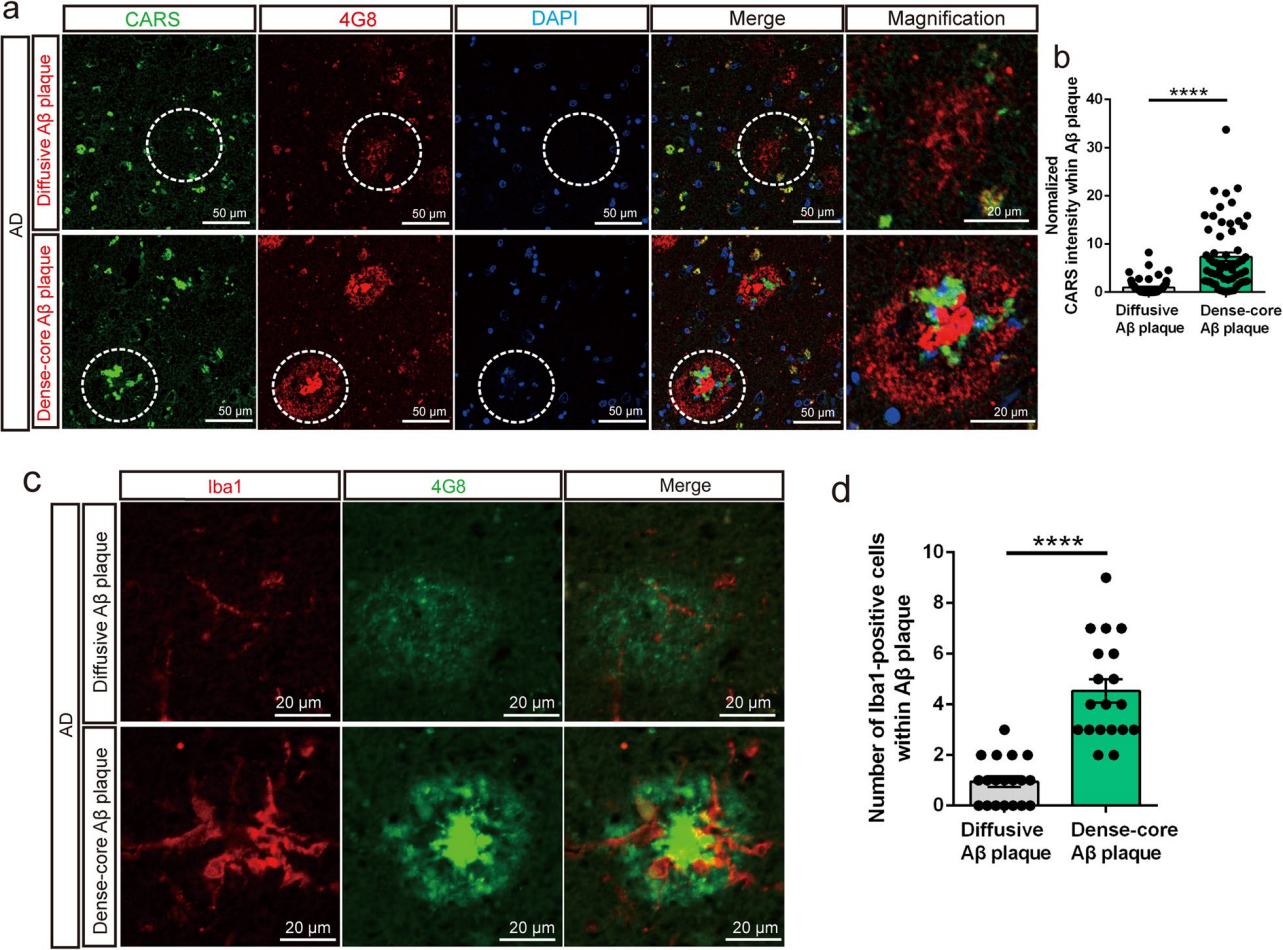
CARS accumulates within dense-core Aβ plaques along with recruitment of ameboid microglia in the post-mortem temporal cortical tissues from AD patients. **a** Representative images showing CARS expression (green) within 4G8 (anti-β-amyloid antibody, red)-labeled diffusive Aβ plaques (upper panels) and dense-core Aβ plaques (lower panels) in the temporal cortical tissues from AD patients. The dashed lines indicate diffusive Aβ plaques (upper panels) and dense-core Aβ plaques (lower panels), and the magnifications are shown in the right panels. **b** Quantification of normalized CARS-ir intensity within the diffusive and the dense-core Aβ plaques in the temporal cortical tissues from three AD subjects. **c** Representative images showing Iba1-labeled microglia (red) within 4G8 (green)-labeled diffusive Aβ plaques (upper panels) and dense-core Aβ plaques (lower panels) in the temporal cortex of the AD subjects. **d** Quantification of microglia within the diffusive and the dense-core Aβ plaques in the temporal lobe cortex from three AD subjects. Data are shown as mean ± SEM; ****P < 0.0001

To determine the association between CARS and Aβ plaques, we performed immunostaining on the postmortem temporal cortex tissues at different Braak stages. The dense-core plaque is a classical plaque characterized by a dense central core of amyloid surrounded by a less compact peripheral halo of amyloid. Immunohistochemistry showed presence of both diffusive Aβ plaques and dense-core Aβ plaques in the patients with AD and the controls (Additional file 3: Fig. S20a). As expected, the dense-core Aβ plaques were the most abundant in the patients with AD at Braak stages V–VI, less abundant in the patients with AD at Braak stages III–IV, and relatively sparse in the controls at Braak stages 0–I (Additional file 3: Fig. S20b). Furthermore, there was a significant positive correlation (*r* = 0.7644, *P* = 0.0009) between the density of dense-core Aβ plaques and the Braak stage in the human temporal cortex (Additional file 3: Fig. S20c). It is noteworthy that CARS accumulated in the dense-core Aβ plaques of the AD patients (Fig. 5a). The CARS-ir signal in the dense-core plaques (normalized CARS intensity: 7.333 ± 0.8758) was nearly sevenfold higher than that in the diffusive plaques (normalized CARS intensity: 1.0 ± 0.1953) (Fig. 5b). We also found clustering of microglia in the dense-core plaques (Fig. 5c). Further analysis showed that the number of microglia within the dense-core Aβ plaques (4.53 ± 0.45) was nearly fivefold higher than that within the diffusive plaques (0.95 ± 0.21) (Fig. 5c, d). Previous studies have demonstrated autofluorescence of the dense-core Aβ plaques under blue‒purple light excitation in both post-mortem brain slices from patients with AD and brain tissues of AD mice [49, 50]. Combining immunofluorescence and Aβ autofluorescence imaging, CARS protein and microglia were both detected within the region of autofluorescence of the dense-core plaques (Additional file 3: Fig. S21). Together, these results suggested that CARS accumulated in the dense-core Aβ plaques with the recruitment of Iba1-positive microglia in the temporal cortex of AD patients.

## Discussion

**Fig. 6:**
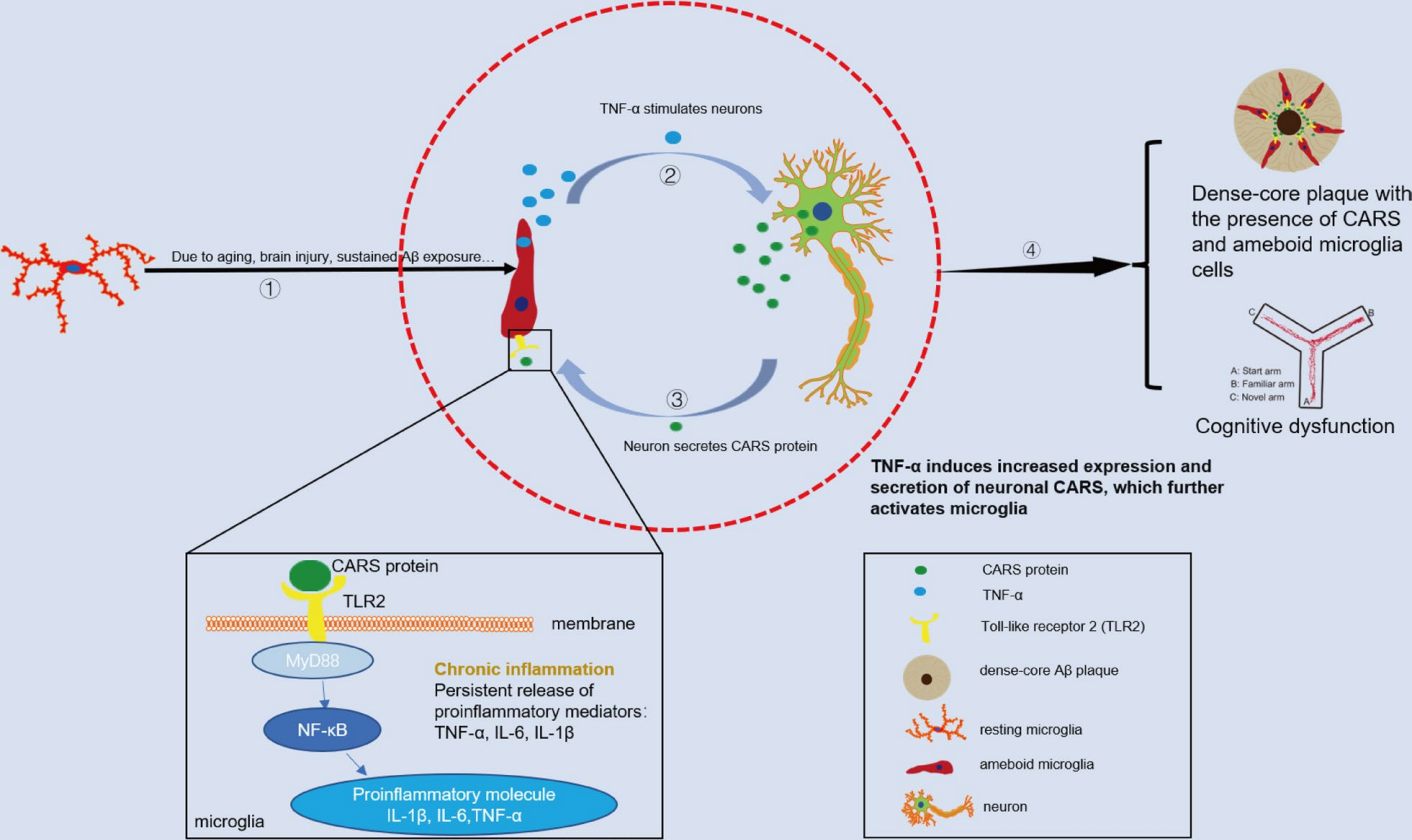
Proposed mechanisms underlying the CARS-induced chronic neuroinflammation in AD. Continuous accumulation of Aβ or other stresses such as oxidative stress can induce chronic neuroinflammation. The increased levels of pro-inflammatory cytokines such as TNF-α stimulate the release of CARS from neurons. Local elevation of CARS can induce additional microglial activation and the release of inflammatory cytokines, which further exacerbate the neuroinflammation cycle, aggravating cognitive impairment

In this study, we identified that CARS, a member of the ARS family, may play a key role in the pathogenesis of AD by regulating the inflammatory response of microglia. We found an increased protein level of CARS in the temporal cortex of AD patients. Overexpression of CARS in the hippocampal neurons induced memory impairment in the C57BL/6J mice and aggravated the memory deficits in APP/PS1 mice, accompanied by activation of microglia and the TLR2/MyD88 signaling pathway. Furthermore, in vitro experiments showed that CARS exacerbated a positive feedback loop of microglial inflammatory response. More specifically, CARS treatment induced chemotaxis, promoting the production of pro-inflammatory cytokines and the activation of the TLR2/MyD88 pathway in mouse microglia BV-2 cells. Treatment of SHSY5Y cells with TNF-α, a proinflammatory cytokine, caused an increase in the expression and secretion of CARS. These results indicated that CARS may contribute to a vicious cycle of chronic neuroinflammation in AD (Fig. 6). Importantly, increased TLR2/MyD88 proteins were also observed in the postmortem temporal cortex tissues from AD patients. Morphological data revealed accumulation of CARS protein within the dense-core Aβ plaques, accompanied by the recruitment of ameboid microglia in AD. Our findings uncover a previously unknown role of neuronal CARS in microglial activation and the pathogenesis of AD, providing insights into novel biomarkers and therapeutic targets for the disease.

As a catalytic enzyme with secondary functions, CARS is implicated in a variety of human diseases, including neurodegenerative diseases [26, 30, 51]. Noticeably, we found that the intensity of CARS-ir in the human temporal cortex was positively correlated with aging. Advanced age is the greatest risk factor for AD [52]. Proteomic investigations have revealed that the balance between protein synthesis and degradation is perturbed during the aging process [53]. It has been reported that proteins associated with oxidative stress and chronic neuroinflammation, as well as other nonfunctional proteins, accumulate and induce neurotoxic effects during aging [54–57]. The important roles of ARSs in neurodegenerative diseases have been increasingly reported in recent studies [15]. In the present study, both the mRNA expression and the protein level of CARS were increased in the temporal cortex of patients with AD, evidencing the potential association between CARS and AD pathogenesis. Interestingly, at Braak stages V–VI, the CARS protein level and mRNA expression showed differential changes. In fact, many proteins implicated in AD, such as tau, TMEM119, alpha4 and alpha7 neuronal nicotinic receptors, exhibit inconsistent changes between mRNA expression and protein level, which may be attributed to the post-transcriptional regulations [58–60]. A recent study has shown that the intron retention (IR) of *CARS* is significantly increased in AD [61]. Moreover, many studies have indicated that the increased IR events lead to decreased efficiency of specific splicing, which may result in premature termination during translation and an imbalance between mRNA and protein homeostasis [61–63].

In the present study, the serum level of CARS was also significantly elevated in the patients with AD. Similarly, an increase in the multisynthetase complex of ARSs, aminoacyl-tRNA synthetase complex-interacting multifunctional protein 1 (AIMP1), was also found in the blood of the AD patients [64]. Interestingly, the AIMP1 level in human blood was negatively associated with the global cognitive function while positively associated with the degree of medial temporal lobe atrophy [64]. Furthermore, our results showed that the serum level of CARS was significantly elevated in the subjects with mild-to-moderate AD compared with those with severe AD, which was consistent with the results of CARS in the post-mortem temporal cortical tissues from AD patients. A previous study has also shown increased expression of some ARS genes at the early Braak stages of AD [22]. Together, these findings provide evidence for the activation of CARS and the ARS family at the early stages of AD.

We showed that the overexpression of CARS in hippocampal neurons induced memory impairment and remarkable activation of microglia in the hippocampus of C57BL/6J mice. Furthermore, the levels of the pro-inflammatory cytokines IL-1β, IL-6, and TNF-α were increased, while the level of the anti-inflammatory cytokine IL-10 was decreased in the hippocampus of CARS-overexpressing mice. Proinflammatory cytokines, such as IL-1β and TNF-α, have been reported to induce neuroinflammation and neurotoxicity in the brain and result in cognitive decline [65, 66]. Therefore, here the cognitive deficits induced by CARS overexpression may be attributed to the activated microglia and the increased levels of pro-inflammatory cytokines. Another study confirmed that CARS could activate immune responses via specific interactions with TLR2/6 on antigen-presenting cells [27]. TLRs (except TLR3) require MyD88 for downstream signaling [67, 68]. Our results showed that neuronal overexpression of CARS induced activation of the TLR2/MyD88 signaling pathway in the hippocampus of C57BL/6J mice. Consistently, the levels of TLR2 and MyD88 in the post-mortem temporal cortex from AD patients were also significantly elevated. A variety of danger-associated molecular pattern molecules, including Aβ, are involved in the elevation of TLR2 in AD patient brains [69]. Moreover, the MyD88 signaling pathway is not restricted to TLR2 activation, since other TLRs and the IL-1 receptor can activate MyD88 signaling as well [70]. Thus, the potential role of other danger-associated molecular pattern molecules in the upregulation of TLR2 and MyD88 in the brains of AD patients cannot be excluded. Furthermore, the activation of the TLR2/MyD88 signaling pathway by CARS was also confirmed in vitro. Treatment of mouse microglial BV-2 cells with CARS increased the levels of IL-1β, IL-6 and TNF-α and induced activation of the TLR2/MyD88 signaling pathway and upregulation of p-AKT, p-JNK, p-ERK and p-P38. Importantly, CARS served as a potent chemoattractant for BV-2 cells, suggesting that CARS may promote microglial migration towards the inflamed site, further exacerbating or augmenting inflammatory responses. In addition, a previous study showed that CARS is secreted by HCT116 cells under endoplasmic reticulum and inflammatory stresses, but not under growth factor or cytokine treatment conditions [27]. Similarly, our results showed that CARS was secreted by SHSY5Y cells treated with TNF-α, indicating that the pro-inflammatory cytokine TNF-α contributes to CARS secretion and exacerbates neuroinflammation.

A large amount of evidence indicates that inflammation contributes to Aβ pathology in AD [71, 72]. Dense-core Aβ plaques are one of the major components of AD neuropathology [72]. Importantly, the number of dense-core plaques is positively correlated with the severity of AD neuropathological changes and cognitive decline [73, 74]. The positive correlation between the density of dense-core plaques and Braak stage in AD found in our study demonstrates that advanced dementia corresponds with high levels of AD neuropathological changes. The accumulation of CARS within dense-core Aβ plaques along with the recruitment of microglia in the temporal cortex provides morphological evidence for the potential involvement of CARS in the pathogenesis of AD (Fig. 6).

There are some limitations in our study. First, the sample sizes of human serum samples and postmortem brain tissues were small. Therefore, the expression level of CARS in AD deserves further investigation with a larger sample size. Second, it should be noted that the results from SHSY5Y cells can not sufficiently represent the neuronal CARS secretion. Further studies using cultured primary neurons or HT-22 cells are needed. Third, questions such as how pro-inflammatory factors regulate the expression of CARS in neurons, whether CARS affects neuronal activity, and how CARS is secreted from neuronal cells in vivo were not addressed in this study, and remain to be explored in future research. Fourth, the possibility that TLRs other than TLR2 are involved in CARS-induced neuroinflammation cannot be excluded. Further studies are needed to measure the levels of other TLRs, such as TLR4. Last but not least, in future studies, it is important to conduct CARS knockdown experiments in AD mouse models, such as APP/PS1, 3 × Tg-AD and 5 × FAD mice, to clarify the causal relationship between CARS and AD pathology.

## Conclusions

Our results suggest for the first time that neuroinflammation driven by increased neuronal CARS may account for the pathology of AD. These findings provide new insights into the cellular and molecular mechanisms underlying the neuroinflammation in AD.

### Supplementary Information

**Additional file 1: Table S1.** Brain material of patients with AD and control subjects. **Table S2.** Serum material of patients with AD and control subjects.

**Additional file 2: Table S3.** Extended statistical information for Figure 1 to Figure 5. **Table S4.** Extended statistical information for Figure S1 to Figure S20.

**Additional file 3: Figure S1.** Increased CARS mRNA level in the temporal cortex of subjects with AD. **Figure S2.** Elevated serum CARS level in the mild-to-moderate AD patients. **Figure S3.** The CARS-immunoreactivity (ir) in GFAP positive astrocytes in the temporal cortex of subjects with Braak stages. **Figure S4.** The CARS-immunoreactivity in Iba1 positive microglia in the temporal cortex of subjects with Braak stages. **Figure S5.** The background fluorescence in paraffin sections of human temporal cortex. **Figure S6.** The histological map showing area of viral infection in the hippocampus of C57BL/6J mice. **Figure S7.** The unedited western blotting gels for Fig. 2f, 3i, k, m. **Figure S8.** Overexpression of CARS in hippocampal neurons of C57BL/6J mice had no effect upon anxiety-like behaviors and motor function. **Figure S9.** Overexpression of CARS in hippocampal neurons exacerbates memory deficit of APP/PS1 mice. **Figure S10.** The histological map showing area of viral infection in the hippocampus of APP/PS1 mice. **Figure S11.** Neuronal CARS overexpression induces microglial morphologic changes and activates the TLR2/MyD88 pathway in hippocampus of APP/PS1 mice. **Figure S12.** The unedited western blotting gels for Fig. S11g, S11j and S11k. **Figure S13.** Increased protein and mRNA levels of TLR2 and MyD88 in the temporal cortex of subjects with AD. **Figure S14.** Knockdown of CARS in hippocampal neurons dose not affect memory of C57BL/6J mice. **Figure S15.** Knockdown of CARS in hippocampal neurons induces downregulation of the TLR2/MyD88 pathway, while does not affect microglial morphology in hippocampus of C57BL/6J mice. **Figure S16.** The unedited western blotting gels for Fig. S15g, S15i and S15k. **Figure S17.** Purified Flag-tagged CARS. **Figure S18.** The unedited western blotting gels for Fig. 4c, 4d, 4e, 4g and 4h. **Figure S19.** TNF-α treatment results in increased levels of CARS protein expression. **Figure S20.** Increased dense-core Aβ plaques in AD. **Figure S21.** CARS is accumulated within dense-core Aβ plaques along with recruitment of ameboid microglial cells in AD.

## Data Availability

All data supporting the conclusions of this article are included within the article and the additional files.

## Abbreviations

AD: Alzheimer's disease
Aβ: β-Amyloid
ARS: Aminoacyl tRNA synthetase
AIMP1: Aminoacyl-tRNA synthetase complex-interacting multifunctional protein 1
CARS: Cysteinyl-tRNA synthetase
CSF: Cerebrospinal fluid
EPM: Elevated plus maze
IL: Interleukin
MyD88: Myeloid differentiation factor 88
NOR: Novel object recognition test
OFT: Open-field test
TLR2: Toll-like receptor 2
TNF-α: Tumor necrosis factor-α
