# Supplementary material for: Increased cysteinyl-tRNA synthetase drives neuroinflammation in Alzheimer’s disease
Source: Transl Neurodegener. 2024 Jan 8;13:3. doi: 10.1186/s40035-023-00394-6 (PMC10773087; doi:10.1186/s40035-023-00394-6)
Supplement: Supplementary file 1 — Additional file 1: Table S1. Brain material of patients with AD and control subjects. Table S2. Serum material of patients with AD and control subjects. [file 40035_2023_394_MOESM1_ESM.doc]

**Table S1 Brain material of patients with AD and control subjects.**

| **Group** | **Braak**  **Aβ plaque**  **stage** | **Braak NFT stage** | **Sex** | **Age** | **PMD (h)** | **CSF pH** | **Brain**  **weight (g)** | **Cause of death** |
| --- | --- | --- | --- | --- | --- | --- | --- | --- |
| **Control** | | | | | | | | |
| C1 | 0 | 0 | F | 60 | 08:10 | 6.58 | 1310 | Metastasized mammacarcinoma |
| C2 | B | Ⅰ | M | 83 | 04:35 | 6.49 | 1367 | Heart attack |
| C3 | B | Ⅰ | F | 91 | 07:45 | 6.90 | 1054 | Severe Decompensation Cordis |
| C4 | B | Ⅰ | F | 92 | 07:00 | 6.45 | 1193 | Possible urosepsis |
| C5 | A | Ⅰ | M | 93 | 07:40 | 6.20 | 1155 | Heart failure |
| C6 | NA | 0 | M | 16 | 19:20 | 7.19 | 960 | Acute leukemia |
| C7 | NA | 0 | F | 22 | 13:25 | 6.2 | 1197 | Osteosarcoma (stage IV) with metastasations to the pelvic cavity |
| C8 | NA | 0 | M | 27 | 07:35 | 7.33 | 1410 | Schizophrenia |
| C9 | NA | 0 | M | 28 | 09:43 | 6.64 | 1093 | Left frontal lobe occupation, cardiopulmonary arrest |
| C10 | NA | 0 | M | 32 | 08:56 | 6.94 | 1238 | Acute heart failure, left ventricular occupation, noninfectious multiple organ dysfunction syndrome, arrhythmia |
| C11 | NA | 0 | M | 32 | 03:40 | 6.5 | 1222 | Chronic respiratory failure |
| C12 | NA | 0 | M | 34 | 09:43 | 6.22 | 1366.9 | Multiple organ failure |
| C13 | NA | 0 | F | 39 | 07:01 | 6.5 | 1220.3 | Mreast carcinoma |
| C14 | NA | 0 | F | 39 | 09:00 | 6.68 | 1369 | Cancer |
| C15 | NA | NA | F | 40 | 10:21 | 4.92 | 1373.5 | Cardiac failure, colorectal carcinoma with metastasations to the lungs, liver, abdominal and pelvic |
| C16 | A | NA | F | 41 | 13:30 | NA | 1263 | NA |
| C17 | NA | 0 | M | 41 | 05:36 | 6.51 | 1421 | Carcinoma of the lungs with metastasations to the bone, pleura, lymph gland and brain |
| C18 | NA | NA | F | 47 | 04:20 | 6.11 | 1217.3 | Gastric carcinoma |
| C19 | NA | 0 | M | 50 | 03:40 | 6.34 | 1430 | Colorectal carcinoma |
| C20 | NA | 0 | F | 51 | 05:31 | 7.11 | 985 | Decompensated cirrhosis after hepatitis B, chronic liver failure |
| C21 | NA | NA | M | 51 | NA | NA | NA | Spontaneous intracerebral hemorrhage, temporal lobe hematoma |
| C22 | 0 | 0 | F | 55 | 07:30 | NA | 1260 | NA |
| C23 | NA | NA | F | 60 | 05:30 | 7.07 | 1215 | NA |
| C24 | NA | NA | M | 61 | 08:00 | NA | NA | Carcinoma of the lungs |
| C25 | A | NA | M | 65 | 05:45 | 6.55 | 1390 | NA |
| C26 | NA | NA | F | 66 | 07:30 | NA | NA | Heart failure, coronary heart disease |
| C27 | A | 0 | M | 68 | 05:50 | 6.5 | 1340 | NA |
| C28 | NA | NA | F | 73 | 07:00 | NA | NA | Carcinoma of the rectum |
| C29 | A | 0 | F | 75 | 09:10 | 6.57 | 1305 | NA |
| C30 | NA | NA | F | 76 | 04:45 | 6.4 | 1140 | NA |
| C31 | A | 0 | F | 78 | 07:10 | 6.32 | 1120 | NA |
| C32 | NA | NA | M | 81 | 04:44 | 6.24 | 1228 | Acute hepatic failure, bladder cancer with metastasations to the bone, septicemia |
| C33 | NA | NA | M | 81 | 02:00 | NA | NA | Gastric antral carcinoma |
| C34 | NA | NA | M | 84 | 17:30 | 6.63 | NA | Hepatitis B |
| C35 | NA | NA | M | 87 | 08:00 | 7.11 | 1255 | Pancreas carcinoma with metastasations to the liver, mesenterium, mesocolon and diaphragma |
| **Mild-to-moderate AD** | | | | | | | | |
| AD1 | NA | Ⅳ | F | 86 | 03:20 | 7.14 | 995 | Cardiac infarction, dyspnea |
| AD2 | C | Ⅳ | M | 88 | 05:00 | 6.45 | 1296 | Physical decline |
| AD3 | B | Ⅲ | F | 90 | 06:05 | 6.12 | 1255 | Possible infection |
| AD4 | C | Ⅳ | M | 92 | 08:25 | 6.14 | 1117 | Heart failure |
| AD5 | 0 | Ⅲ | F | 92 | 06:35 | 6.12 | 1305 | Heart failure |
| **Severe AD** | | | | | | | | |
| AD6 | C | Ⅴ | M | 59 | 07:45 | 6.29 | 1171 | Dehydration |
| AD7 | C | Ⅵ | F | 70 | 08:15 | 6.30 | 876 | Aspiration pneumonia |
| AD8 | NA | Ⅴ | F | 85 | 03:10 | 6.90 | 1044 | Bronchopneumonia |
| AD9 | C | Ⅴ | F | 85 | 06:10 | 6.65 | 1003 | Dehydration |
| AD10 | NA | Ⅴ | M | 95 | 07:00 | 6.18 | 1143 | Gastrointestinal bleeding |

Abbreviations: AD, Alzheimer’s disease; Aβ, β-amyloid; CSF, cerebrospinal fluid; F, female; M, male; NFT, neurofibrillary tangle; PMD, post-mortem delay; NA, not available.

**Table S2 Serum material of patients with AD and control subjects.**

| **Group** | **Age** | **Sex** | **Clinical diagnosis** | **MMSE** | **MoCA** |
| --- | --- | --- | --- | --- | --- |
| Control-1 | 78 | M | non-AD control | 29 | NA |
| Control-2 | 79 | F | non-AD control | 28 | NA |
| Control-3 | 79 | F | non-AD control | 29 | NA |
| Control-4 | 82 | M | non-AD control | 29 | NA |
| Control-5 | 86 | F | non-AD control | 23 | NA |
| Control-6 | 86 | F | non-AD control | NA | 23 |
| Control-7 | 87 | F | non-AD control | 28 | NA |
| Control-8 | 87 | M | non-AD control | NA | 25 |
| Control-9 | 88 | M | non-AD control | NA | 23 |
| Control-10 | 89 | F | non-AD control | 26 | NA |
| Control-11 | 89 | M | non-AD control | NA | 24 |
| Control-12 | 89 | M | non-AD control | NA | 24 |
| Control-13 | 89 | M | non-AD control | NA | 24 |
| Control-14 | 91 | M | non-AD control | NA | 24 |
| Control-15 | 91 | F | non-AD control | NA | 24 |
| Control-16 | 91 | M | non-AD control | 22 | NA |
| Control-17 | 92 | F | non-AD control | 25 | NA |
| Control-18 | 93 | M | non-AD control | 27 | NA |
| Control-19 | 95 | M | non-AD control | NA | 24 |
| Mild-to-moderate AD-1 | 68 | F | mild AD | 26 | 21 |
| Mild-to-moderate AD-2 | 82 | F | mild AD | NA | NA |
| Mild-to-moderate AD-3 | 85 | F | moderate AD | NA | NA |
| Mild-to-moderate AD-4 | 86 | F | moderate AD | NA | NA |
| Mild-to-moderate AD-5 | 89 | F | moderate AD | NA | NA |
| Mild-to-moderate AD-6 | 89 | F | moderate AD | NA | NA |
| Mild-to-moderate AD-7 | 90 | F | mild AD | NA | NA |
| Mild-to-moderate AD-8 | 91 | F | moderate AD | NA | NA |
| Mild-to-moderate AD-9 | 102 | F | moderate AD | NA | NA |
| Mild-to-moderate AD-10 | 102 | F | moderate AD | NA | NA |
| Severe AD-1 | 64 | F | severe AD | NA | NA |
| Severe AD-2 | 69 | M | severe AD | NA | NA |
| Severe AD-3 | 72 | M | severe AD | NA | NA |
| Severe AD-4 | 76 | F | severe AD | NA | NA |
| Severe AD-5 | 78 | F | severe AD | NA | NA |
| Severe AD-6 | 79 | F | severe AD | NA | NA |
| Severe AD-7 | 79 | F | severe AD | NA | NA |
| Severe AD-8 | 83 | F | severe AD | NA | NA |
| Severe AD-9 | 88 | M | severe AD | NA | NA |
| Severe AD-10 | 91 | M | severe AD | NA | NA |
| Severe AD-11 | 91 | F | severe AD | NA | NA |
| Severe AD-12 | 92 | F | severe AD | NA | NA |
| Severe AD-13 | 71 | M | severe AD | NA | NA |
|  |  |  |  |  |  |
| Severe AD-14 | 72 | M | severe AD | NA | NA |
| Severe AD-15 | 73 | M | severe AD | NA | NA |
| Severe AD-16 | 76 | M | severe AD | NA | NA |
| Severe AD-17 | 83 | F | severe AD | NA | NA |
| Severe AD-18 | 84 | F | severe AD | NA | NA |
| Severe AD-19 | 84 | M | severe AD | NA | NA |
| Severe AD-20 | 84 | M | severe AD | NA | NA |
| Severe AD-21 | 86 | F | severe AD | NA | NA |
| Severe AD-22 | 89 | F | severe AD | NA | NA |
| Severe AD-23 | 90 | F | severe AD | NA | NA |
| Severe AD-24 | 91 | F | severe AD | NA | NA |
| Severe AD-25 | 93 | F | severe AD | NA | NA |
| Severe AD-26 | 96 | F | severe AD | NA | NA |

Abbreviations: F, female; M, male; AD, Alzheimer’s disease; MMSE, Mini-Mental State Examination; MoCA, Montreal Cognitive Assessment Scale; NA, not available.
