# Supplementary material for: Increased cysteinyl-tRNA synthetase drives neuroinflammation in Alzheimer’s disease
Source: Transl Neurodegener. 2024 Jan 8;13:3. doi: 10.1186/s40035-023-00394-6 (PMC10773087; doi:10.1186/s40035-023-00394-6)
Supplement: Supplementary file 2 — Additional file 2: Table S3. Extended statistical information for Figure 1 to Figure 5. Table S4. Extended statistical information for Figure S1 to Figure S20. [file 40035_2023_394_MOESM2_ESM.doc]

**Table S3. Extended statistical information for Fig. 1 to Fig. 5.**

| **Figure Panel** | **n/group** | **Primary statistic** | **Post-hoc test** | **Comparison** | **p value** | **Statistic** |
| --- | --- | --- | --- | --- | --- | --- |
| **Fig. 1a** | n = 35 subjects | Pearson correlation test |  |  | 0.006 | r = 0.4557 |
| **Fig. 1c** | Braak 0–Ⅰ, n = 15 slices from 5 subjects  Braak Ⅲ–Ⅳ, n = 15 slices from 5 subjects  Braak Ⅴ–Ⅵ, n = 15 slices from 5 subjects | One-way ANOVA | Bonferroni’s  multiple comparison | Main effect of group  Braak 0–Ⅰ vs. Braak Ⅲ–Ⅳ  Braak 0–Ⅰ vs. Braak Ⅴ–Ⅵ  Braak Ⅲ–Ⅳ vs. Braak Ⅴ–Ⅵ | < 0.0001  < 0.0001  0.0394  0.0006 | F(2,42) = 22.63 |
| **Fig. 1e** | Braak 0–Ⅰ, n = 5 subjects  Braak Ⅲ–Ⅳ, n = 5 subjects  Braak Ⅴ–Ⅵ, n = 5 subjects | One-way ANOVA | Bonferroni’s  multiple comparison | Main effect of group  Braak 0–Ⅰ vs. Braak Ⅲ–Ⅳ  Braak 0–Ⅰ vs. Braak Ⅴ–Ⅵ  Braak Ⅲ–Ⅳ vs. Braak Ⅴ–Ⅵ | 0.0001  0.0001  0.0484  0.0107 | F(2,12) = 20.64 |
| **Fig. 1g** | Braak 0–Ⅰ, n = 15 slices from 5 subjects  Braak Ⅲ–Ⅳ, n = 15 slices from 5 subjects  Braak Ⅴ–Ⅵ, n = 15 slices from 5 subjects | One-way ANOVA | Bonferroni’s  multiple comparison | Main effect of group  Braak 0–Ⅰ vs. Braak Ⅲ–Ⅳ  Braak 0–Ⅰ vs. Braak Ⅴ–Ⅵ  Braak Ⅲ–Ⅳ vs. Braak Ⅴ–Ⅵ | < 0.0001  0.4330  < 0.0001  < 0.0001 | F(2,42) = 27.31 |
| **Fig. 1h** | Braak 0–Ⅰ, n = 75 neurons from 5 subjects  Braak Ⅲ–Ⅳ, n = 75 neurons from 5 subjects  Braak Ⅴ–Ⅵ, n = 75 neurons from 5 subjects | One-way ANOVA | Bonferroni’s  multiple comparison | Main effect of group  Braak 0–Ⅰ vs. Braak Ⅲ–Ⅳ  Braak 0–Ⅰ vs. Braak Ⅴ–Ⅵ  Braak Ⅲ–Ⅳ vs. Braak Ⅴ–Ⅵ | < 0.0001  < 0.0001  < 0.0001  0.0578 | F(2,222) = 29.54 |
| **Fig. 2b** | Cortex, n = 14 ROIs from 3 mice  Hippocampus, n = 14 ROIs from 3 mice | Unpaired t-test |  |  | 0.3054 | t26 = 1.046 |
| **Fig. 2f** | Control, n = 4 mice  Overexpression, n = 4 mice | Unpaired t-test with Welch’s correction |  |  | 0.0033 | t3.217 = 7.864 |
| **Fig. 2g** | Control, n = 6 mice  Overexpression, n = 7 mice | Unpaired t-test |  |  | 0.0144 | t11 = 2.902 |
| **Fig. 2h** | Control, n = 6 mice  Overexpression, n = 7 mice | Unpaired t-test |  |  | 0.0089 | t11 = 3.174 |
| **Fig. 2i** | Control, n = 6 mice  Overexpression, n = 7 mice | Unpaired t-test |  |  | 0.0303 | t11 = 2.485 |
| **Fig. 2j** | Control, n = 6 mice  Overexpression, n = 7 mice | Unpaired t-test |  |  | 0.0385 | t11 = 2.349 |
| **Fig. 2l** | Control, n = 6 mice  Overexpression, n = 7 mice | Two-way RM ANOVA | Bonferroni’s  multiple comparison | Group × object interaction  Main effect of object  Control: Familiar vs. Novel  Overexpression: Familiar vs. Novel | 0.0012  0.1740  0.0046  0.1141 | F(1,11) = 18.84  F(1,11) = 2.112 |
| **Fig. 2m** | Control, n = 6 mice  Overexpression, n = 7 mice | Unpaired t-test with Welch’s correction |  |  | 0.0006 | t7.812 = 5.478 |
| **Fig. 2n** | Control, n = 6 mice  Overexpression, n = 7 mice | Unpaired t-test |  |  | 0.3781 | t11 = 0.918 |
| **Fig. 3b** | Control, n = 10 ROIs from 2 mice  Overexpression, n = 10 ROIs from 3 mice | Unpaired t-test |  |  | 0.0050 | t18 = 3.198 |
| **Fig. 3c** | Control, n = 44 cells from 2 mice  Overexpression, n = 48 cells from 3 mice | Unpaired t-test |  |  | < 0.0001 | t90 = 9.929 |
| **Fig. 3d** | Control, n = 44 cells from 2 mice  Overexpression, n = 48 cells from 3 mice | Unpaired t-test |  |  | < 0.0001 | t90 = 10.75 |
| **Fig. 3e** | Control, n = 44 cells from 2 mice  Overexpression, n = 48 cells from 3 mice | Unpaired t-test with Welch’s correction |  |  | < 0.0001 | t75.76 = 9.200 |
| **Fig. 3f** | Control, n = 44 cells from 2 mice  Overexpression, n = 48 cells from 3 mice | Unpaired t-test with Welch’s correction |  |  | < 0.0001 | t78.67 = 8.160 |
| **Fig. 3g** | Control, n = 42 cells from 2 mice  Overexpression, n = 43 cells from 3 mice | Two-way RM ANOVA |  | Group × distance interaction  Main effect of group | < 0.0001  < 0.0001 | F(59,4897) = 25.04  F(1,83) = 114.3 |
| **Fig. 3j** | TLR2: Control, n = 3 mice  Overexpression, n = 3 mice  MyD88: Control, n = 3 mice  Overexpression, n = 3 mice  p-NF-κB: Control, n = 3 mice  Overexpression, n = 3 mice | Unpaired t-test  Unpaired t-test  Unpaired t-test |  |  | 0.0008  0.0225  0.0119 | t4 = 9.075  t4 = 3.611  t4 = 4.379 |
| **Fig. 3l** | p-AKT: Control, n = 3 mice  Overexpression, n = 3 mice  p-JNK: Control, n = 3 mice  Overexpression, n = 3 mice  p-ERK: Control, n = 3 mice  Overexpression, n = 3 mice  p-P38: Control, n = 3 mice  Overexpression, n = 3 mice | Unpaired t-test  Unpaired t-test  Unpaired t-test  Unpaired t-test |  |  | 0.0056  0.0004  0.0005  0.0067 | t4 = 5.418  t4 = 10.95  t4 = 10.46  t4 = 5.155 |
| **Fig. 3n** | IL-6: Control, n = 3 mice  Overexpression, n = 3 mice  TNF-α: Control, n = 3 mice  Overexpression, n = 3 mice  IL-1β: Control, n = 3 mice  Overexpression, n = 3 mice  IL-10: Control, n = 3 mice  Overexpression, n = 3 mice | Unpaired t-test  Unpaired t-test  Unpaired t-test  Unpaired t-test |  |  | 0.0083  0.0008  0.0137  0.0030 | t4 = 4.861  t4 = 9.174  t4 = 4.199  t4 = 6.428 |
| **Fig. 4b** | Vehicle, n = 9 ROIs from three replications  CARS, n = 9 ROIs from three replications | Unpaired t-test with Welch’s correction |  |  | < 0.0001 | t8.197 = 33.23 |
| **Fig. 4f** | IL-6: Vehicle, n = 3 replications  CARS, n = 3 replications  IL-1β: Vehicle, n = 3 replications  CARS, n = 3 replications  IL-10: Vehicle, n = 3 replications  CARS, n = 3 replications  TNF-α: Vehicle, n = 6 replications  CARS, n = 6 replications  TLR2: Vehicle, n = 6 replications  CARS, n = 6 replications  p-NF-κB: Vehicle, n = 3 replications  CARS, n = 3 replications  MyD88: Vehicle, n = 3 replications  CARS, n = 3 replications | Unpaired t-test  Unpaired t-test  Unpaired t-test  Unpaired t-test  Unpaired t-test  Unpaired t-test  Unpaired t-test |  |  | 0.0042  0.0290  0.0120  0.0007  < 0.0001  0.0001  0.0035 | t4 = 5.858  t4 = 3.334  t4 = 4.365  t10 = 4.782  t10 = 8.068  t4 = 14.36  t4 = 6.164 |
| **Fig. 4i** | p-AKT: Vehicle, n = 3 replications  CARS, n = 3 replications  p-JNK: Vehicle, n = 3 replications  CARS, n = 3 replications  p-ERK: Vehicle, n = 3 replications  CARS, n = 3 replications  p-P38: Vehicle, n = 3 replications  CARS, n = 3 replications | Unpaired t-test  Unpaired t-test  Unpaired t-test  Unpaired t-test |  |  | < 0.0001  0.0043  0.0367  0.0034 | t4 = 17.02  t4 = 5.840  t4 = 3.087  t4 = 6.235 |
| **Fig. 5b** | Diffusive Aβ plaques, n = 62 ROIs  Dense-core Aβ plaques, n = 61 ROIs | Unpaired t-test with Welch’s correction |  |  | < 0.0001 | t65.95 = 7.058 |
| **Fig. 5d** | Diffusive Aβ plaques, n = 19 ROIs  Dense-core Aβ plaques, n = 19 ROIs | Unpaired t-test with Welch’s correction |  |  | < 0.0001 | t25.28 = 7.150 |

**Table S4. Extended statistical information for Fig. S1 to Fig. S20.**

| **Figure Panel** | **n/group** | **Primary statistic** | **Post-hoc test** | **Comparison** | **p value** | **Statistic** |
| --- | --- | --- | --- | --- | --- | --- |
| **Fig. S1** | Braak 0–Ⅰ, n = 5 subjects  Braak Ⅲ–Ⅳ, n = 5 subjects  Braak Ⅴ–Ⅵ, n = 5 subjects | One-way ANOVA | Bonferroni’s multiple comparison | Main effect of group  Braak 0–Ⅰ vs. Braak Ⅲ–Ⅳ  Braak 0–Ⅰ vs. Braak Ⅴ–Ⅵ  Braak Ⅲ–Ⅳ vs. Braak Ⅴ–Ⅵ | 0.0483  0.4181  0.0482  0.7414 | F(2,12) = 3.942 |
| **Fig. S2** | Control, n = 19 subjects  Mild-to-moderate AD, n = 10 subjects  Severe AD, n = 26 subjects | One-way ANOVA | Bonferroni’s multiple comparison | Main effect of group  Control vs. Mild-to-moderate AD  Control vs. Severe AD  Mild-to-moderate AD vs. Severe AD | 0.0176  0.0178  > 0.9999  0.0480 | F(2,52) = 4.370 |
| **Fig. S3b** | Braak 0–Ⅰ, n = 15 ROIs from 5 subjects  Braak Ⅲ–Ⅳ, n = 15 ROIs from 5 subjects  Braak Ⅴ–Ⅵ, n = 15 ROIs from 5 subjects | One-way ANOVA | Bonferroni’s multiple comparison | Main effect of group  Braak 0–Ⅰ vs. Braak Ⅲ–Ⅳ  Braak 0–Ⅰ vs. Braak Ⅴ–Ⅵ  Braak Ⅲ–Ⅳ vs. Braak Ⅴ–Ⅵ | < 0.0001  < 0.0001  0.0008  0.5227 | F(2,42) = 15.44 |
| **Fig. S3c** | Braak 0–Ⅰ, n = 15 ROIs from 5 subjects  Braak Ⅲ–Ⅳ, n =15 ROIs from 5 subjects  Braak Ⅴ–Ⅵ, n = 15 ROIs from 5 subjects | One-way ANOVA | Bonferroni’s multiple comparison | Main effect of group  Braak 0–Ⅰ vs. Braak Ⅲ–Ⅳ  Braak 0–Ⅰ vs. Braak Ⅴ–Ⅵ  Braak Ⅲ–Ⅳ vs. Braak Ⅴ–Ⅵ | < 0.0001  < 0.0001  < 0.0001  0.0423 | F(2,42) = 29.15 |
| **Fig. S4b** | Braak 0–Ⅰ, n = 15 ROIs from 5 subjects  Braak Ⅲ–Ⅳ, n = 15 ROIs from 5 subjects  Braak Ⅴ–Ⅵ, n = 15 ROIs from 5 subjects | One-way ANOVA | Bonferroni’s multiple comparison | Main effect of group  Braak 0–Ⅰ vs. Braak Ⅲ–Ⅳ  Braak 0–Ⅰ vs. Braak Ⅴ–Ⅵ  Braak Ⅲ–Ⅳ vs. Braak Ⅴ–Ⅵ | 0.0975  0.1021  >0.9999  0.5085 | F(2,42) = 2.461 |
| **Fig. S4c** | Braak 0–Ⅰ, n = 15 ROIs from 5 subjects  Braak Ⅲ–Ⅳ, n = 15 ROIs from 5 subjects  Braak Ⅴ–Ⅵ, n = 15 ROIs from 5 subjects | One-way ANOVA | Bonferroni’s multiple comparison | Main effect of group  Braak 0–Ⅰ vs. Braak Ⅲ–Ⅳ  Braak 0–Ⅰ vs. Braak Ⅴ–Ⅵ  Braak Ⅲ–Ⅳ vs. Braak Ⅴ–Ⅵ | 0.1005  0.8500  0.8113  0.0993 | F(2,42) = 2.428 |
| **Fig. S6b** | AAV-EGFP, n = 14 slices from 3 mice  AAV-CARS, n = 15 slices from 3 mice | Unpaired t-test |  |  | 0.5898 | t27 = 0.5456 |
| **Fig. S8a** | Control, n = 6 mice  Overexpression, n = 7 mice | Unpaired t-test |  |  | 0.1541 | t11 = 1.531 |
| **Fig. S8b** | Control, n = 6 mice  Overexpression, n = 7 mice | Unpaired t-test |  |  | 0.3346 | t11 = 1.009 |
| **Fig. S8c** | Control, n = 6 mice  Overexpression, n = 7 mice | Unpaired t-test |  |  | 0.5418 | t11 = 0.630 |
| **Fig. S8d** | Control, n = 6 mice  Overexpression, n = 7 mice | Unpaired t-test |  |  | 0.7252 | t11 = 0.361 |
| **Fig. S8e** | Control, n = 6 mice  Overexpression, n = 7 mice | Unpaired t-test |  |  | 0.6694 | t11 = 0.439 |
| **Fig. S8f** | Control, n = 6 mice  Overexpression, n = 7 mice | Unpaired t-test |  |  | 0.3621 | t11 = 0.951 |
| **Fig. S9d** | Control, n = 3 mice  Overexpression, n = 3 mice | Unpaired t-test |  |  | 0.0038 | t4 = 6.019 |
| **Fig. S9e** | Control, n = 6 mice  Overexpression, n = 6 mice | Unpaired t-test |  |  | 0.0188 | t10 = 2.801 |
| **Fig. S9f** | Control, n = 6 mice  Overexpression, n = 6 mice | Unpaired t-test |  |  | 0.0771 | t10 = 1.970 |
| **Fig. S9g** | Control, n = 6 mice  Overexpression, n = 6 mice | Unpaired t-test |  |  | 0.0488 | t10 = 2.242 |
| **Fig. S9h** | Control, n = 6 mice  Overexpression, n = 6 mice | Unpaired t-test |  |  | 0.0165 | t10 = 2.877 |
| **Fig. S9j** | Control, n = 6 mice  Overexpression, n = 6 mice | Two-way RM ANOVA | Bonferroni’s multiple comparison | Group × object interaction  Main effect of object  Control: Familiar vs. Novel  Overexpression: Familiar vs. Novel | 0.8526  0.9406  > 0.9999  > 0.9999 | F(1,10) = 0.0364  F(1,10) = 0.0058 |
| **Fig. S9k** | Control, n = 6 mice  Overexpression, n = 6 mice | Unpaired t-test |  |  | 0.9085 | t10 = 0.1178 |
| **Fig. S9l** | Control, n = 6 mice  Overexpression, n = 6 mice | Unpaired t-test |  |  | 0.8095 | t10 = 0.2476 |
| **Fig. S9n** | Control, n = 6 mice  Overexpression, n = 6 mice | Unpaired t-test |  |  | 0.4838 | t10 = 0.7271 |
| **Fig. S9o** | Control, n = 6 mice  Overexpression, n = 6 mice | Unpaired t-test |  |  | 0.1767 | t10 =1.454 |
| **Fig. S9p** | Control, n = 6 mice  Overexpression, n = 6 mice | Unpaired t-test |  |  | 0.8212 | t10 = 0.2320 |
| **Fig. S9q** | Control, n = 6 mice  Overexpression, n = 6 mice | Unpaired t-test |  |  | 0.7248 | t10 = 0.3621 |
| **Fig. S9r** | Control, n = 6 mice  Overexpression, n = 6 mice | Unpaired t-test |  |  | 0.8217 | t10 = 0.2313 |
| **Fig. S9s** | Control, n = 6 mice  Overexpression, n = 6 mice | Unpaired t-test |  |  | 0.6049 | t10 = 0.5342 |
| **Fig. S10b** | AAV-EGFP, n = 15 slices from 3 mice  AAV-CARS, n = 15 slices from 3 mice | Unpaired t-test |  |  | 0.9961 | t28 = 0.0049 |
| **Fig. S11b** | Control, n = 69 cells from 2 mice  Overexpression, n = 53 cells from 2 mice | Unpaired t-test with Welch’s correction |  |  | < 0.0001 | t116.8 = 12.74 |
| **Fig. S11c** | Control, n = 69 cells from 2 mice  Overexpression, n = 53 cells from 2 mice | Unpaired t-test with Welch’s correction |  |  | < 0.0001 | t117.4 = 13.98 |
| **Fig. S11d** | Control, n = 69 cells from 2 mice  Overexpression, n = 53 cells from 2 mice | Unpaired t-test with Welch’s correction |  |  | < 0.0001 | t116.2 = 14.18 |
| **Fig. S11e** | Control, n = 69 cells from 2 mice  Overexpression, n = 53 cells from 2 mice | Unpaired t-test with Welch’s correction |  |  | < 0.0001 | t100.1 = 13.69 |
| **Fig. S11f** | Control, n = 69 cells from 2 mice  Overexpression, n = 53 cells from 2 mice | Two-way RM ANOVA |  | Group × distance interaction  Main effect of group | < 0.0001  < 0.0001 | F(49,5880) = 21.66  F(1,120) = 128.3 |
| **Fig. S11h** | TLR2: Control, n = 3 mice  Overexpression, n = 3 mice  MyD88: Control, n = 3 mice  Overexpression, n = 3 mice  p-NF-κB: Control, n = 3 mice  Overexpression, n = 3 mice | Unpaired t-test  Unpaired t-test  Unpaired t-test |  |  | < 0.0001  0.0463  0.0908 | t4 = 41.97  t4 = 2.852  t4 = 2.218 |
| **Fig. S11j** | p-AKT: Control, n = 3 mice  Overexpression, n = 3 mice  p-JNK: Control, n = 3 mice  Overexpression, n = 3 mice  p-ERK: Control, n = 3 mice  Overexpression, n = 3 mice  p-P38: Control, n = 3 mice  Overexpression, n = 3 mice | Unpaired t-test  Unpaired t-test  Unpaired t-test  Unpaired t-test with Welch’s correction |  |  | 0.2446  0.0057  0.0207  0.0496 | t4 = 1.363  t4 = 5.396  t4 = 3.706  t2.031 = 4.257 |
| **Fig. S11l** | IL-6: Control, n = 3 mice  Overexpression, n = 3 mice  IL-1β: Control, n = 3 mice  Overexpression, n = 3 mice  TNF-α: Control, n = 3 mice  Overexpression, n = 3 mice | Unpaired t-test  Unpaired t-test  Unpaired t-test |  |  | 0.7252  0.9453  0.0494 | t4 = 0.3772  t4 = 0.0731  t4 = 2.788 |
| **Fig. S13b** | TLR2: Non-AD, n = 5 subjects  AD, n = 5 subjects  MyD88: Non-AD, n = 5 subjects  AD, n = 5 subjects | Unpaired t-test  Unpaired t-test with Welch’s correction |  |  | 0.0126  0.0014 | t8 = 3.202  t4.091 = 7.644 |
| **Fig. S13c** | TLR2: Non-AD, n = 5 subjects  AD, n = 5 subjects  MyD88: Non-AD, n = 5 subjects  AD, n = 5 subjects | Unpaired t-test with Welch’s correction  Unpaired t-test with Welch’s correction |  |  | 0.0262  0.0179 | t4.594 = 3.229  t4.624 = 3.592 |
| **Fig. S14d** | Scramble, n = 3 mice  ShCARS, n = 3 mice | Unpaired t-test |  |  | 0.0134 | t4 = 4.226 |
| **Fig. S14e** | Scramble, n = 5 mice  ShCARS, n = 5 mice | Unpaired t-test |  |  | 0.2931 | t8 = 1.125 |
| **Fig. S14f** | Scramble, n = 5 mice  ShCARS, n = 5 mice | Unpaired t-test |  |  | 0.5004 | t8 = 0.7057 |
| **Fig. S14g** | Scramble, n = 5 mice  ShCARS, n = 5 mice | Unpaired t-test |  |  | 0.3916 | t8 = 0.9057 |
| **Fig. S14h** | Scramble, n = 5 mice  ShCARS, n = 5 mice | Unpaired t-test |  |  | 0.3081 | t8 = 0.1.089 |
| **Fig. S14j** | Scramble, n = 5 mice  ShCARS, n = 5 mice | Two-way RM ANOVA | Bonferroni’s multiple comparison | Group × object interaction  Main effect of object  Scramble: Familiar vs. Novel  shRNA: Familiar vs. Novel | 0.8788  0.0011  0.0131  0.0181 | F(1,8) = 0.0248  F(1,8) = 24.99 |
| **Fig. S14k** | Scramble, n = 5 mice  ShCARS, n = 5 mice | Unpaired t-test |  |  | 0.7087 | t8 = 0.3872 |
| **Fig. S14l** | Scramble, n = 5 mice  ShCARS, n = 5 mice | Unpaired t-test |  |  | 0.3176 | t8 = 1.066 |
| **Fig. S14n** | Scramble, n = 5 mice  ShCARS, n = 5 mice | Unpaired t-test |  |  | 0.9148 | t8 = 0.1104 |
| **Fig. S14o** | Scramble, n = 5 mice  ShCARS, n = 5 mice | Unpaired t-test |  |  | 0.5059 | t8 = 0.6965 |
| **Fig. S14p** | Scramble, n = 5 mice  ShCARS, n = 5 mice | Unpaired t-test |  |  | 0.7638 | t8 = 0.3109 |
| **Fig. S14q** | Scramble, n = 5 mice  ShCARS, n = 5 mice | Unpaired t-test |  |  | 0.6668 | t8 = 0.4469 |
| **Fig. S14r** | Scramble, n = 5 mice  ShCARS, n = 5 mice | Unpaired t-test |  |  | 0.5564 | t8 = 0.6138 |
| **Fig. S14s** | Scramble, n = 5 mice  ShCARS, n = 5 mice | Unpaired t-test |  |  | 0.2258 | t8 = 1.312 |
| **Fig. S15b** | Scramble, n = 28 cells from 2 mice  ShCARS, n = 33 cells from 2 mice | Unpaired t-test with Welch’s correction |  |  | 0.4049 | t49.94 = 0.8401 |
| **Fig. S15c** | Scramble, n = 28 cells from 2 mice  ShCARS, n = 33 cells from 2 mice | Unpaired t-test with Welch’s correction |  |  | 0.6163 | t56.21 = 0.5039 |
| **Fig. S15d** | Scramble, n = 28 cells from 2 mice  ShCARS, n = 33 cells from 2 mice | Unpaired t-test |  |  | 0.1509 | t59 = 1.455 |
| **Fig. S15e** | Scramble, n = 28 cells from 2 mice  ShCARS, n = 33 cells from 2 mice | Unpaired t-test |  |  | 0.7715 | t59 = 0.2918 |
| **Fig. S15f** | Scramble, n = 28 cells from 2 mice  ShCARS, n = 33 cells from 2 mice | Two-way RM ANOVA |  | Group × distance interaction  Main effect of group | < 0.0001  0.5976 | F(59,3481) = 2.952  F(1,59) = 0.2816 |
| **Fig. S15h** | TLR2: Scramble, n = 3 mice  ShCARS, n = 3 mice  MyD88: Scramble, n = 3 mice  ShCARS, n = 3 mice  p-NF-κB: Scramble, n = 3 mice  ShCARS, n = 3 mice | Unpaired t-test  Unpaired t-test  Unpaired t-test |  |  | 0.0965  0.0232  0.0934 | t4 = 2.164  t4 = 3.577  t4 = 2.192 |
| **Fig. S15j** | p-AKT: Scramble, n = 3 mice  ShCARS n, n = 3 mice  p-JNK: Scramble, n = 3 mice  ShCARS, n = 3 mice  p-ERK: Scramble, n = 3 mice  ShCARS, n = 3 mice  p-P38: Scramble, n = 3 mice  ShCARS, n = 3 mice | Unpaired t-test with Welch’s correction  Unpaired t-test  Unpaired t-test  Unpaired t-test |  |  | 0.0087  0.8296  0.3901  0.5495 | t2.094 = 9.871  t4 = 0.2297  t4 = 0.9629  t4 = 0.6529 |
| **Fig. S15l** | IL-6: Scramble, n = 3 mice  ShCARS, n = 3 mice  TNF-α: Scramble, n = 3 mice  ShCARS, n = 3 mice | Unpaired t-test  Unpaired t-test |  |  | 0.3896  0.8408 | t4 = 0.964  t4 = 0.2143 |
| **Fig. S19b** | 0 ng/ml, n = 4 replications  1 ng/ml, n = 4 replications  5 ng/ml, n = 4 replications  10 ng/ml, n = 4 replications | One-way ANOVA | Bonferroni’s multiple comparison | Main effect of group  0 ng/ml vs. 1 ng/ml  0 ng/ml vs. 5 ng/ml  0 ng/ml vs. 10 ng/ml | < 0.0001  0.0113  < 0.0001  < 0.0001 | F(3,12) = 45.21 |
| **Fig. S19d** | 0 hrs, n = 3 replications  3 hrs, n = 3 replications  6 hrs, n = 3 replications  12 hrs, n = 3 replications  24 hrs, n = 3 replications | One-way ANOVA | Bonferroni’s multiple comparison | Main effect of group  0 hrs vs. 3 hrs  0 hrs vs. 6 hrs  0 hrs vs. 12 hrs  0 hrs vs. 24 hrs | < 0.0001  0.0016  < 0.0001  < 0.0001  < 0.0001 | F(4,10) = 85.07 |
| **Fig. S20b** | Braak 0–Ⅰ, n = 14 slices from 5 subjects  Braak Ⅲ–Ⅳ, n = 14 slices from 5 subjects  Braak Ⅴ–Ⅵ, n = 14 slices from 5 subjects | One-way ANOVA | Bonferroni’s multiple comparison | Main effect of group  Braak 0–Ⅰ vs. Braak Ⅲ–Ⅳ  Braak 0–Ⅰ vs. Braak Ⅴ–Ⅵ  Braak Ⅲ–Ⅳ vs. Braak Ⅴ–Ⅵ | < 0.0001  0.0085  < 0.0001  0.0004 | F(2,39) = 27.94 |
| **Fig. S20c** | n = 15 subjects | Pearson correlation test |  |  | 0.0009 | r = 0.7644 |
