# Supplementary material for: Increased cysteinyl-tRNA synthetase drives neuroinflammation in Alzheimer’s disease
Source: Transl Neurodegener. 2024 Jan 8;13:3. doi: 10.1186/s40035-023-00394-6 (PMC10773087; doi:10.1186/s40035-023-00394-6)
Supplement: Supplementary file 3 — Additional file 3: Figure S1. Increased CARS mRNA level in the temporal cortex of subjects with AD. Figure S2. Elevated serum CARS level in the mild-to-moderate AD patients. Figure S3. The CARS-immunoreactivity (ir) in GFAP positive astrocytes in the temporal cortex of subjects with Braak stages. Figure S4. The CARS-immunoreactivity in Iba1 positive microglia in the temporal cortex of subjects with Braak stages. Figure S5. The background fluorescence in paraffin sections of human temporal cortex. Figure S6. The histological map showing area of viral infection in the hippocampus of C57BL/6J mice. Figure S7. The unedited western blotting gels for Fig. 2f, 3i, k, m. Figure S8. Overexpression of CARS in hippocampal neurons of C57BL/6J mice had no effect upon anxiety-like behaviors and motor function. Figure S9. Overexpression of CARS in hippocampal neurons exacerbates memory deficit of APP/PS1 mice. Figure S10. The histological map showing area of viral infection in the hippocampus of APP/PS1 mice. Figure S11. Neuronal CARS overexpression induces microglial morphologic changes and activates the TLR2/MyD88 pathway in hippocampus of APP/PS1 mice. Figure S12. The unedited western blotting gels for Fig. S11g, S11j and S11k. Figure S13. Increased protein and mRNA levels of TLR2 and MyD88 in the temporal cortex of subjects with AD. Figure S14. Knockdown of CARS in hippocampal neurons dose not affect memory of C57BL/6J mice. Figure S15. Knockdown of CARS in hippocampal neurons induces downregulation of the TLR2/MyD88 pathway, while does not affect microglial morphology in hippocampus of C57BL/6J mice. Figure S16. The unedited western blotting gels for Fig. S15g, S15i and S15k. Figure S17. Purified Flag-tagged CARS. Figure S18. The unedited western blotting gels for Fig. 4c, 4d, 4e, 4g and 4h. Figure S19. TNF-α treatment results in increased levels of CARS protein expression. Figure S20. Increased dense-core Aβ plaques in AD. Figure S21. CARS is accumulated within dense-core Aβ [file 40035_2023_394_MOESM3_ESM.pdf]

Additional file 3

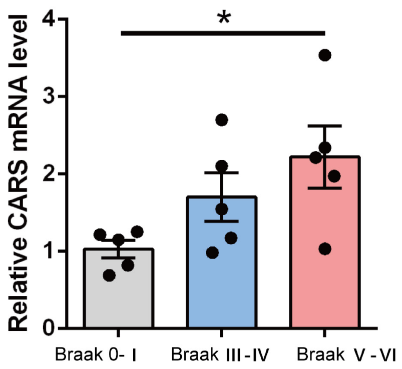

Fig. S1 Increased CARS mRNA level in the temporal cortex of subjects with AD. Relative CARS mRNA level in the temporal cortex of subjects with Braak stages. Data are shown as mean ± SEM; *$p < 0.05$.

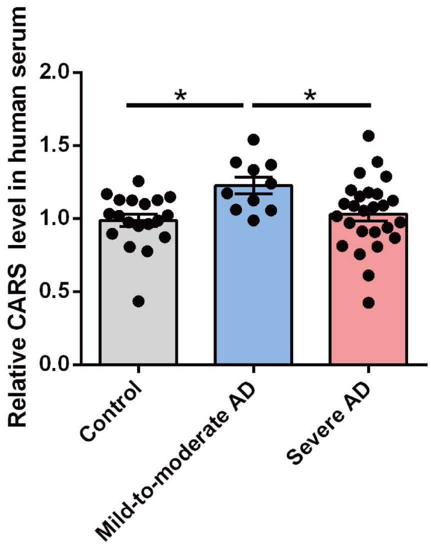

Fig. S2 Elevated serum CARS level in the mild-to-moderate AD patients. CARS level in the serum of AD subjects. Data are shown as mean ± SEM; *$p < 0.05$.

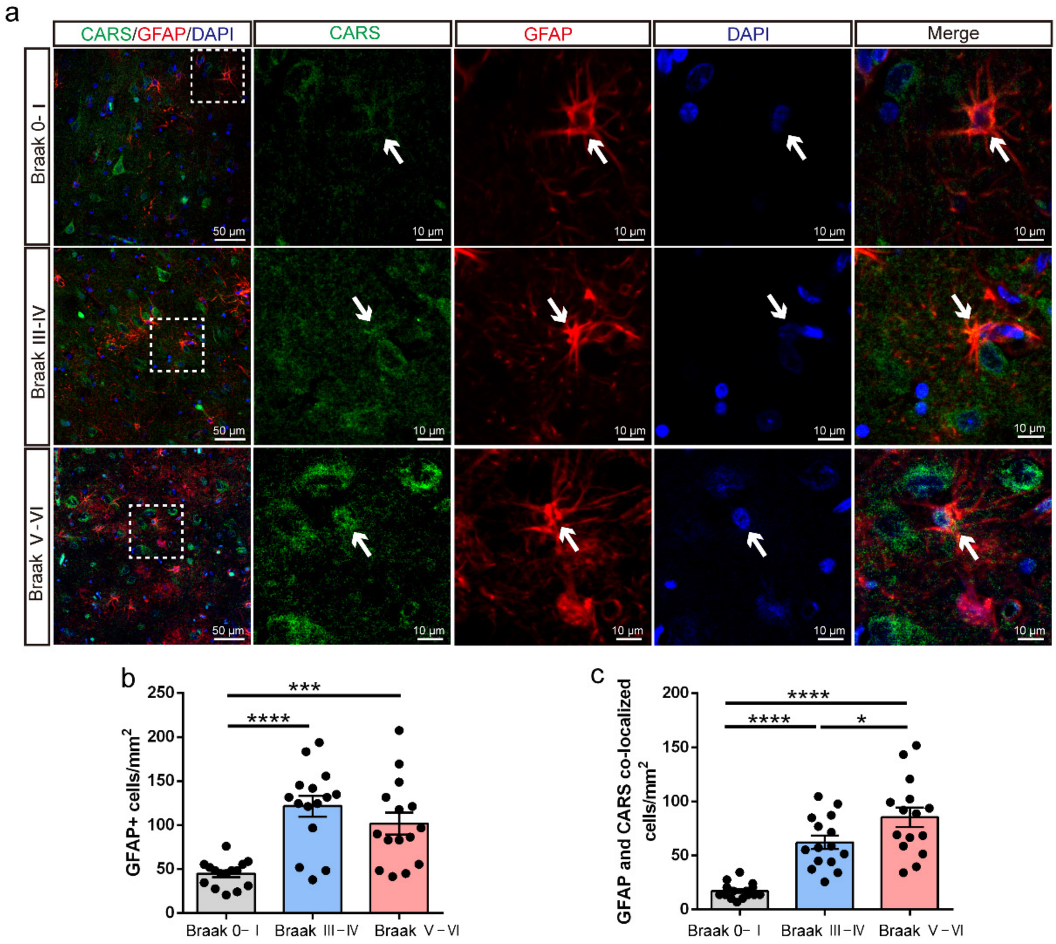

Fig. S3 The CARS-immunoreactivity (ir) in GFAP positive astrocytes in the temporal cortex of subjects with Braak stages. **a** Representative images of CARS-ir (green) and GFAP (red) in the temporal cortex of subjects with Braak stages. Magnifications of white dotted boxes are shown in the right four panels. White arrows denote the co-localization of CARS-ir and GFAP. Scale bars: left panels, 50 μm; right four panels, 10 μm. **b** Quantification of the density of GFAP+ astrocytes in the temporal cortex of subjects with Braak stages. **c** Quantification of the density of CARS and GFAP co-localized cells in the temporal cortex of subjects with Braak stages. Data are shown as mean ± SEM; $*p < 0.05$, $***p < 0.001$, $****p < 0.0001$.

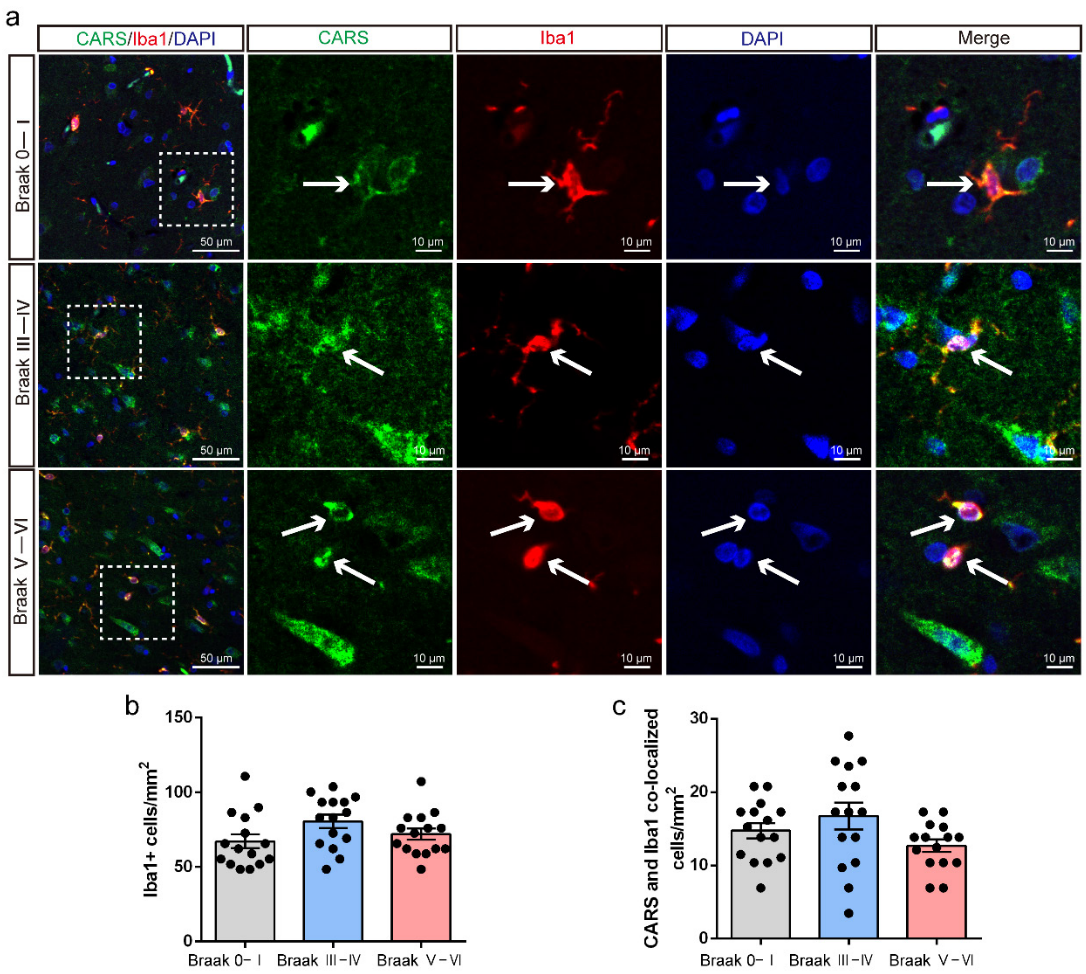

Fig. S4 The CARS-immunoreactivity in Iba1 positive microglia in the temporal cortex of subjects with Braak stages. **a** Representative images of CARS- ir (green) and Iba1 (red) in the temporal cortex of subjects with Braak stages. Magnifications of white dotted boxes are shown in the right four panels. White arrows denote the co-localization of CARS-ir and Iba1. Scale bars: left panels, 50 µm; right four panels, 10 µm. **b** Quantification of the density of Iba1+ microglia in the temporal cortex of subjects with Braak stages. **c** Quantification of the density of CARS and Iba1 co-localized cells in the temporal cortex of subjects with Braak stages. Data are shown as mean ± SEM.

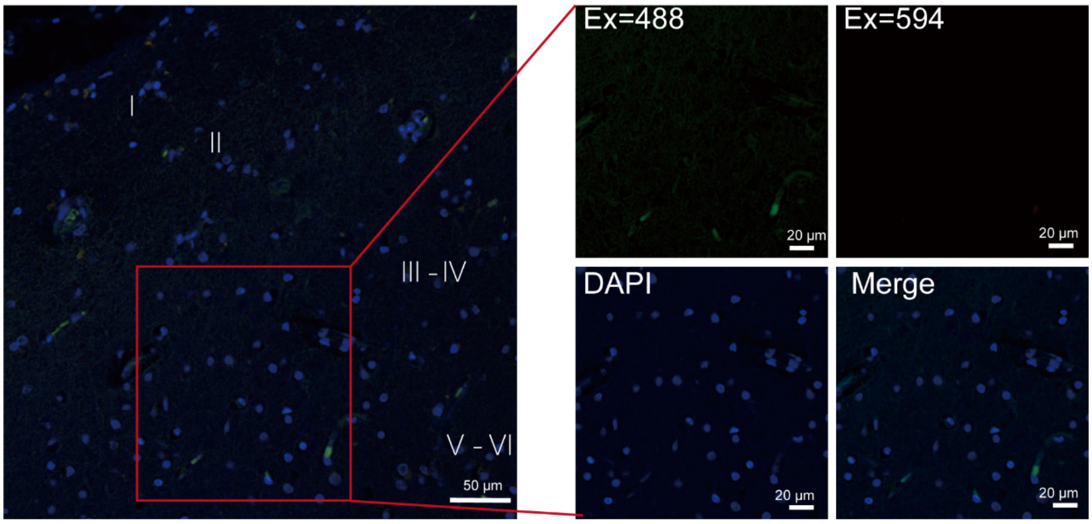

Fig. S5 The background fluorescence in paraffin sections of human temporal cortex. Representative images showing the fluorescence of paraffin sections of human temporal cortex excited 405 nm, 488 nm and 594 nm after staining with Alexa Fluor® 488 AffiniPure Donkey Anti-Rabbit IgG (H+L) antibody and DAPI, respectively. Scale bars: left panel: 50 μm; right four panels: 20 μm.

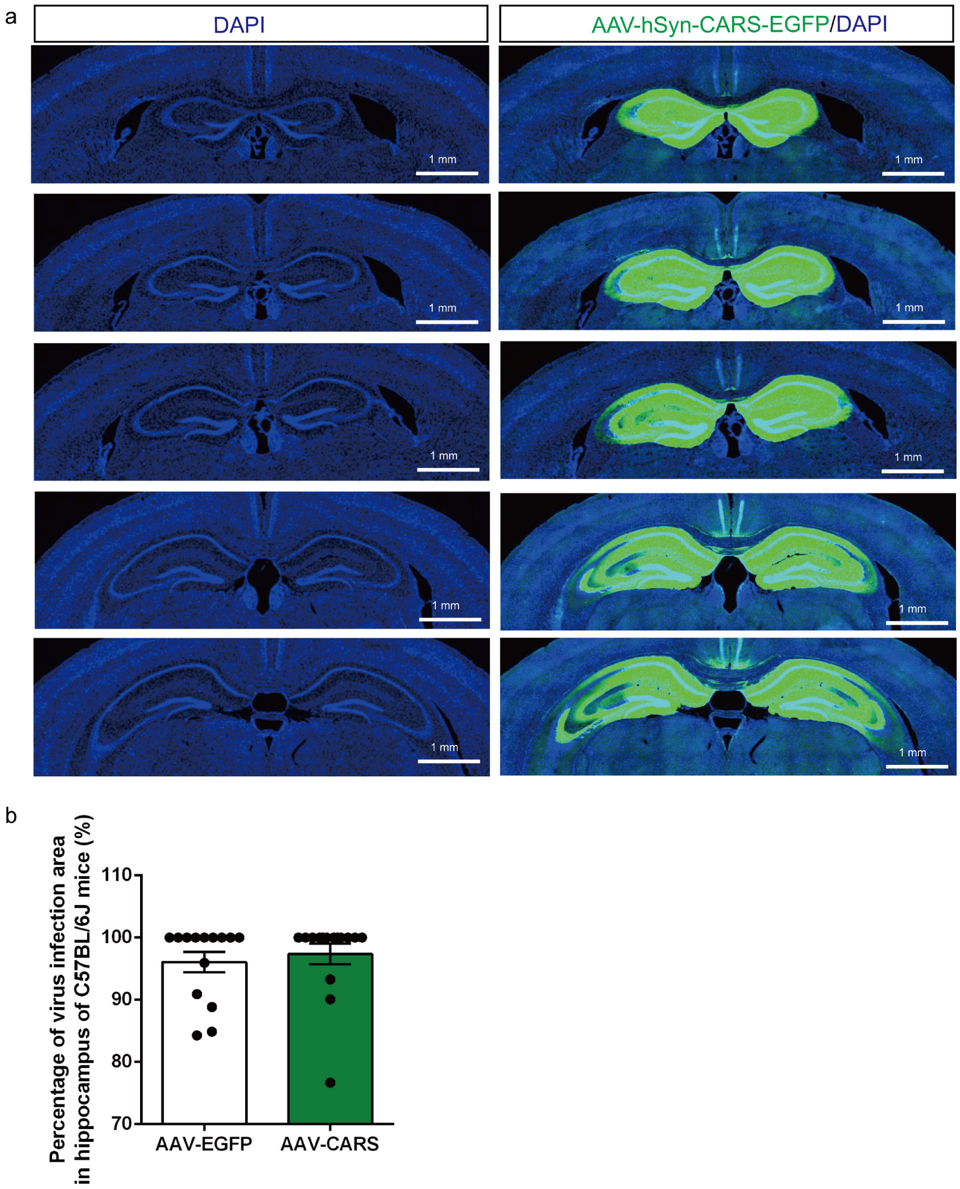

Fig. S6 The histological map showing area of viral infection in the hippocampus of C57BL/6J mice. a Example brain slices from a C57BL/6J mouse injected with AAV-hSyn-CARS-EGFP (AAV-CARS) in the hippocampus (left: DAPI; right: AAV-hSyn-CARS-EGFP and DAPI). Scale bars: 1 mm. **b** Percentage of virus infection area in each. Data are shown as mean ± SEM.

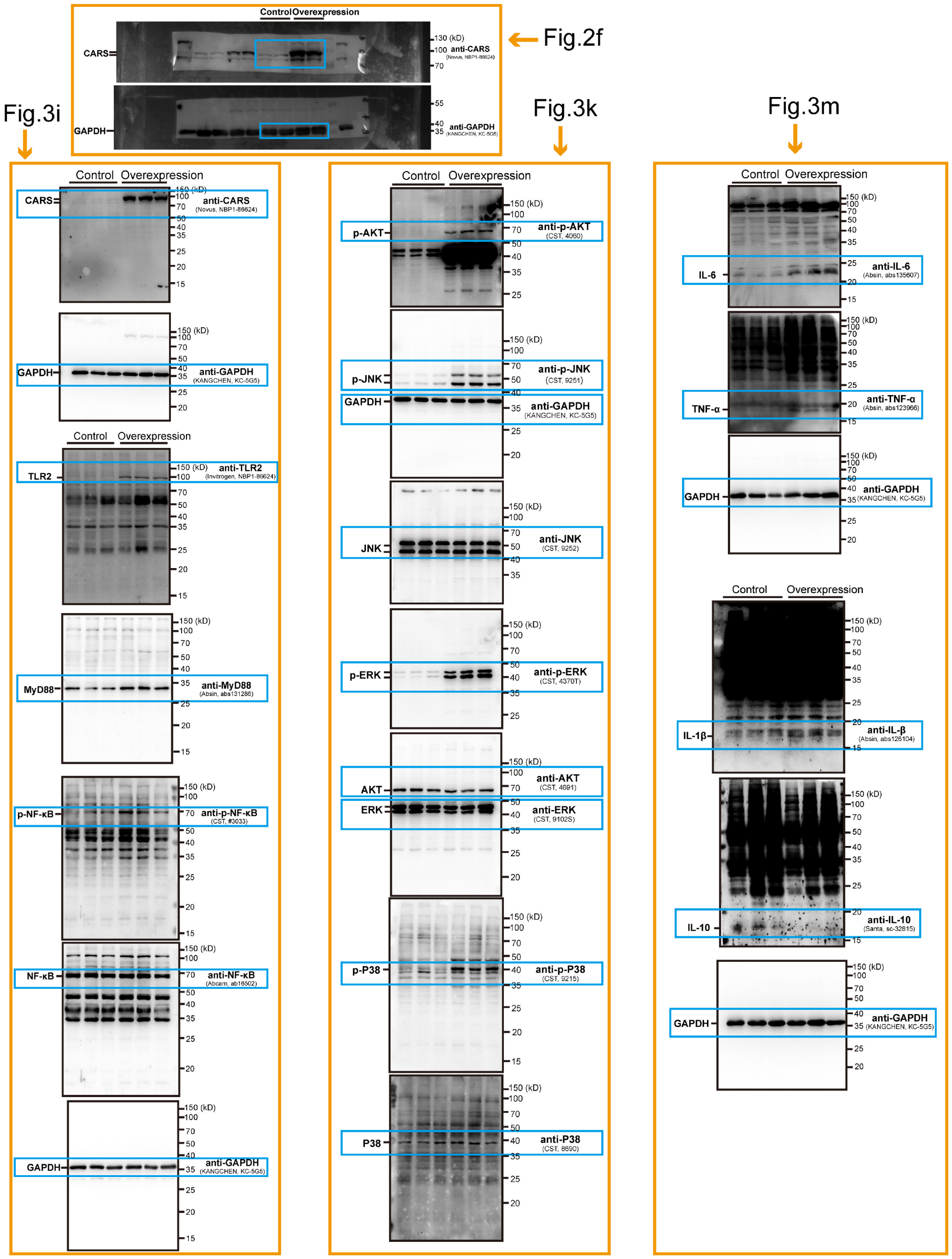

Fig. S7 The unedited western blotting gels for Fig. 2f, 3i, 3k and 3m.

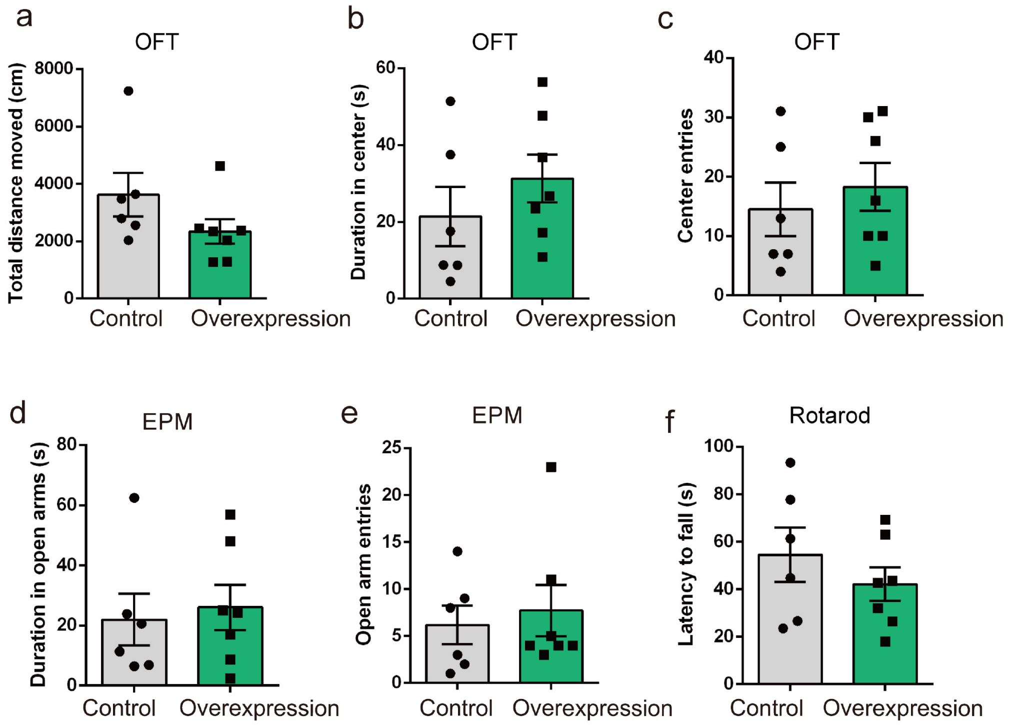

Fig. S8 Overexpression of CARS in hippocampal neurons of C57BL/6J mice had no effect upon anxiety-like behaviors and motor function. a–c The total distance moved (**a**), time spent (**b**) and entries (**c**) in the center area in the open field test (OFT). **d** and **e** The time (**d**) and entries (**e**) in the open arms in the elevated plus maze (EPM) test. **f** The average latency to fall in the rotarod test. Data are shown as mean ± SEM.

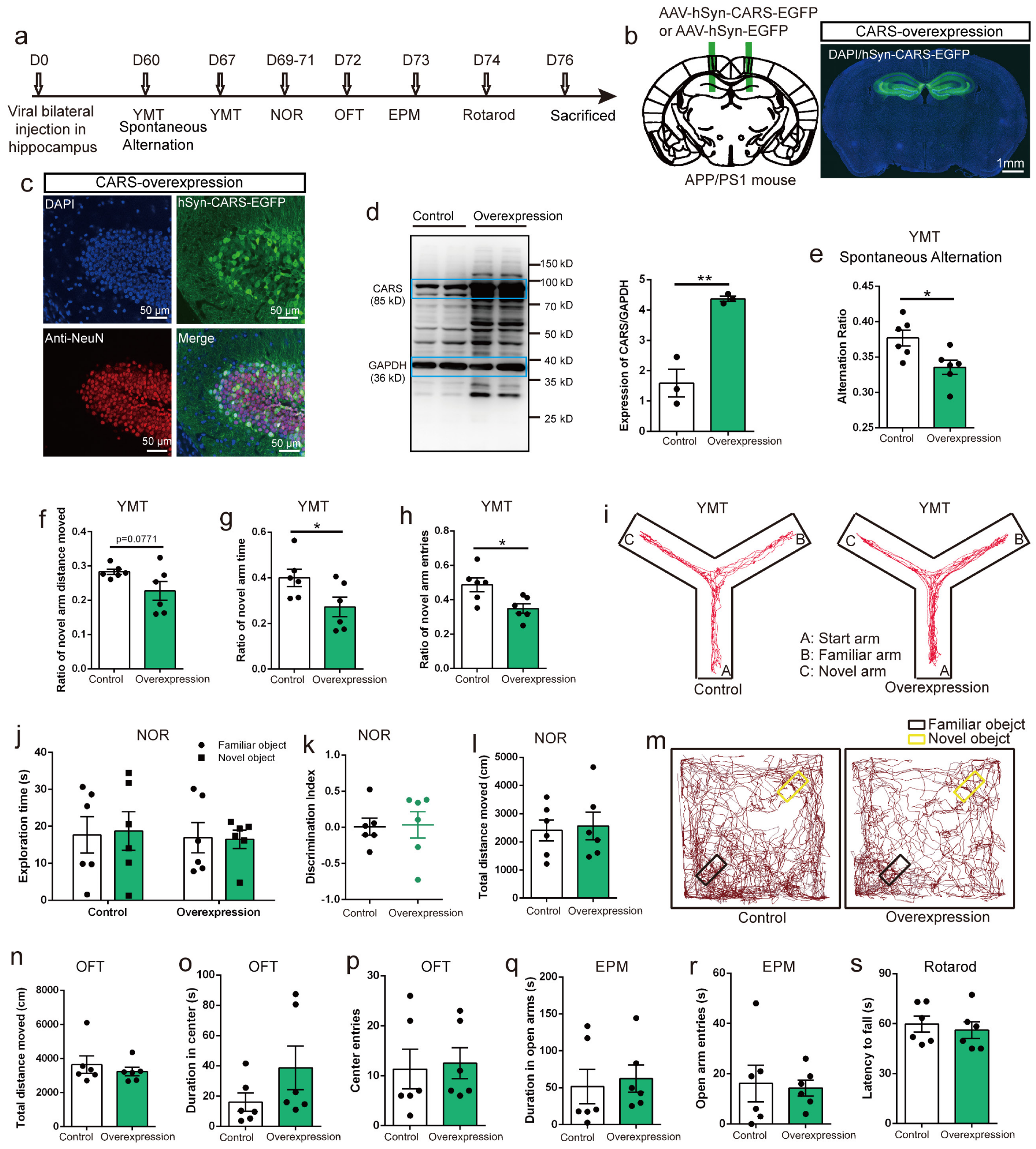

Fig. S9 Overexpression of CARS in hippocampal neurons exacerbates memory deficit of APP/PS1 mice. a The schedule of viral injection and the following behavioral tests. **b** Left: bilateral injection of AAV-hSyn-CARS-EGFP (CARS-overexpression or overexpression) or AAV-hSyn-EGFP (control) into the hippocampus of APP/PS1 mice. Right: a representative image of infected hippocampus; scale bar: 1 mm. **c** Immunostaining of NeuN in the hippocampus after the hSynapsin promoter-driven expression of CARS; scale bars: 50 μm. **d** Left, western blotting of CARS protein expression in the hippocampus of CARS-

overexpression or control mice. Right, quantification of CARS protein expression in the hippocampus. **e** Spontaneous alternation behavior determined by Y-maze test (YMT). **f–h** Ratios of distance moved (**f**), time spent (**g**) and entries (**h**) in the novel arm in the YMT. (**i**) Representative activity traces of control and CARS-overexpression mice in the YMT. **j–l** The exploration time of familiar and novel objects (**j**), discrimination index (**k**) and total distance moved (**l**) in the novel object recognition (NOR) test. **m** Representative activity traces of control and CARS-overexpression mice in the NOR test. **n–p** The total distance moved (**n**), time spent (**o**) and entries (**p**) in the center area in the open field test (OFT). **q** and **r** The time (**q**) and entries (**r**) in the open arms in the elevated plus maze (EPM) test. **s** The average latency to fall in the rotarod test. Data are shown as mean ± SEM; $*p < 0.05$, $**p < 0.01$.

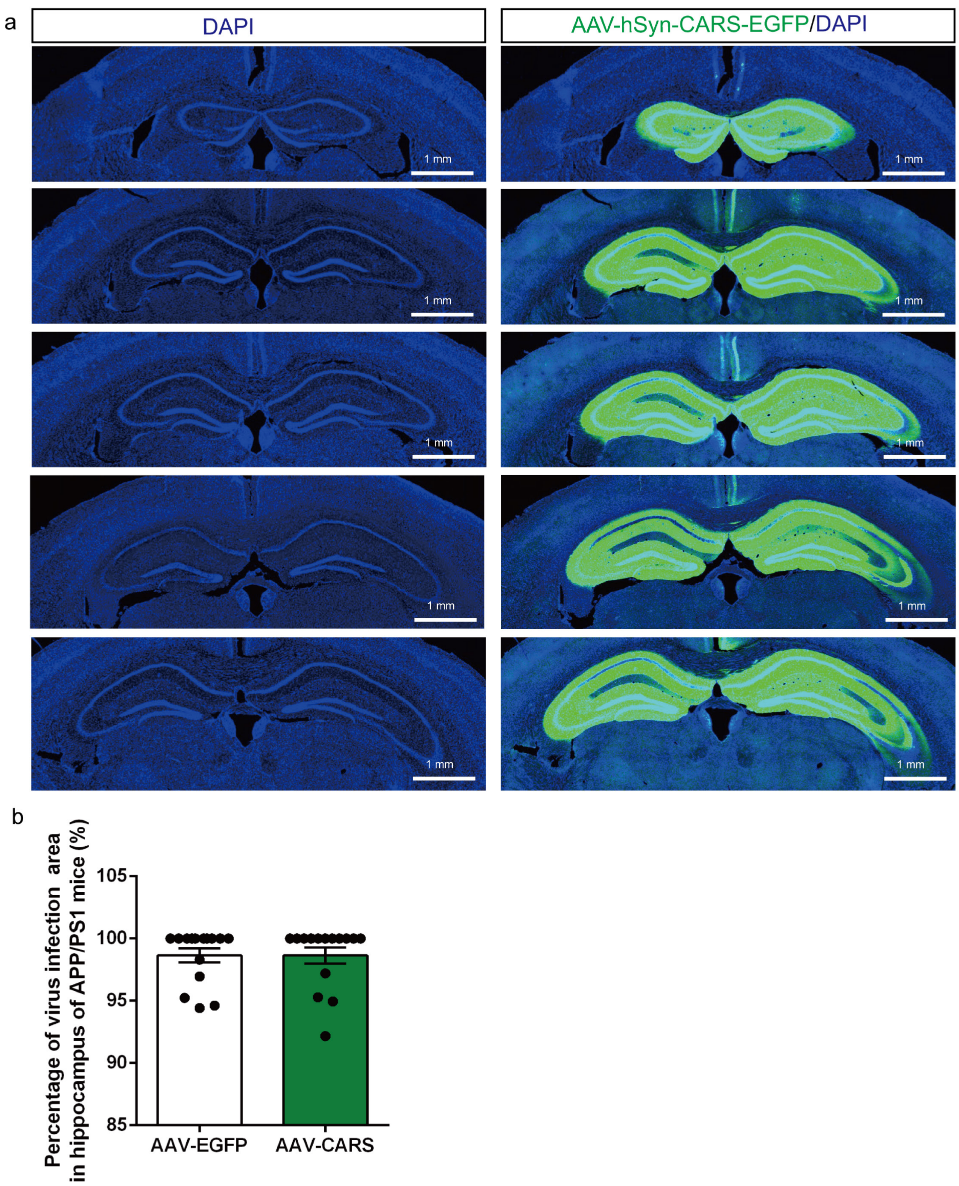

Fig. S10 The histological map showing area of viral infection in the hippocampus of APP/PS1 mice. a Example brain slices from a APP/PS1 mouse injected with AAV-hSyn-CARS-EGFP in the hippocampus (left: DAPI; right: AAV-hSyn-CARS-EGFP and DAPI). Scale bars: 1 mm. **b** Percentage of virus infection area in each hippocampus. Data are shown as mean ± SEM.

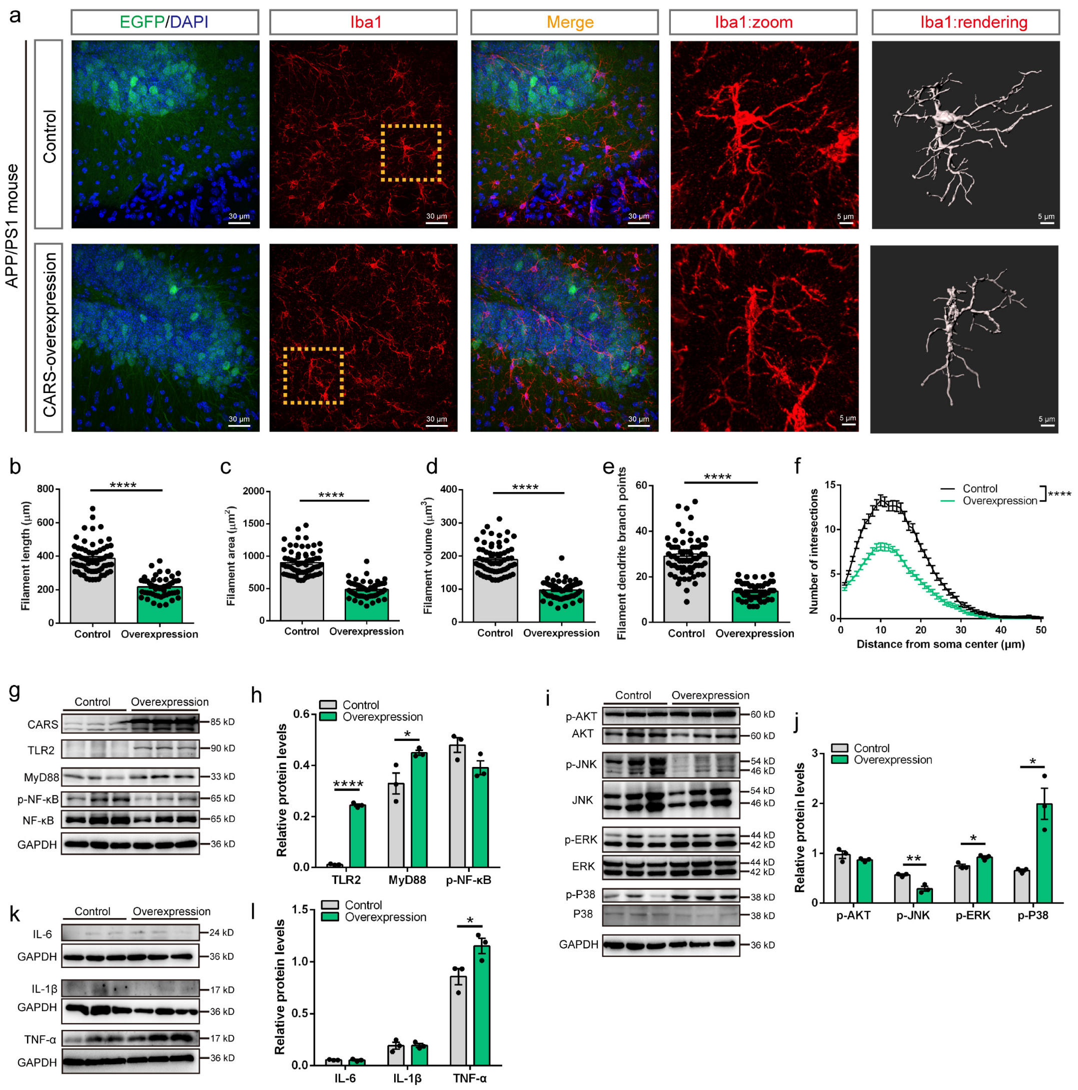

Fig. S11 Neuronal CARS overexpression induces microglial morphologic changes and activates the TLR2/MyD88 pathway in hippocampus of APP/PS1 mice. **a** Representative images of Iba1 immunostaining (red) and three-dimensional (3D) reconstruction (gray) of microglia in the hippocampus from APP/PS1 mice treated with AAV-hSyn-CARS-EGFP (CARS-overexpression or overexpression) or AAV-hSyn-EGFP (control). Zoomed in view and rendering are shown in the right two panels respectively. Scale bars: 30 μm (overview) and 5 μm (zoomed in view and rendering). **b**–**e** Imaris-based automated quantification of Iba1+ microglial filament length (**b**), filament area (**c**), filament volume (**d**), and numbers of dendrite branch points (**e**) in the hippocampus from control and CARS-overexpression APP/PS1 mice. **f** Sholl analysis of microglial morphology in control and

CARS-overexpression APP/PS1 mice. **g** and **h** Representative western blotting images (**g**) and quantification (**h**) of TLR2, MyD88 and p-NF-κB protein expression in the hippocampus of control and CARS-overexpression APP/PS1 mice. **i** and **j** Representative western blotting images (**i**) and quantification (**j**) of p-AKT, p-JNK, p-ERK and p-P38 protein expression in the hippocampus of control and CARS-overexpression APP/PS1 mice. **k** and **l** Representative western blotting images (**k**) and quantification (**l**) of IL-6, IL-1β, and TNF-α protein expression in the hippocampus of control and CARS-overexpression APP/PS1 mice. Data are shown as mean ± SEM; $*p < 0.05$, $**p < 0.01$, $****p < 0.0001$.

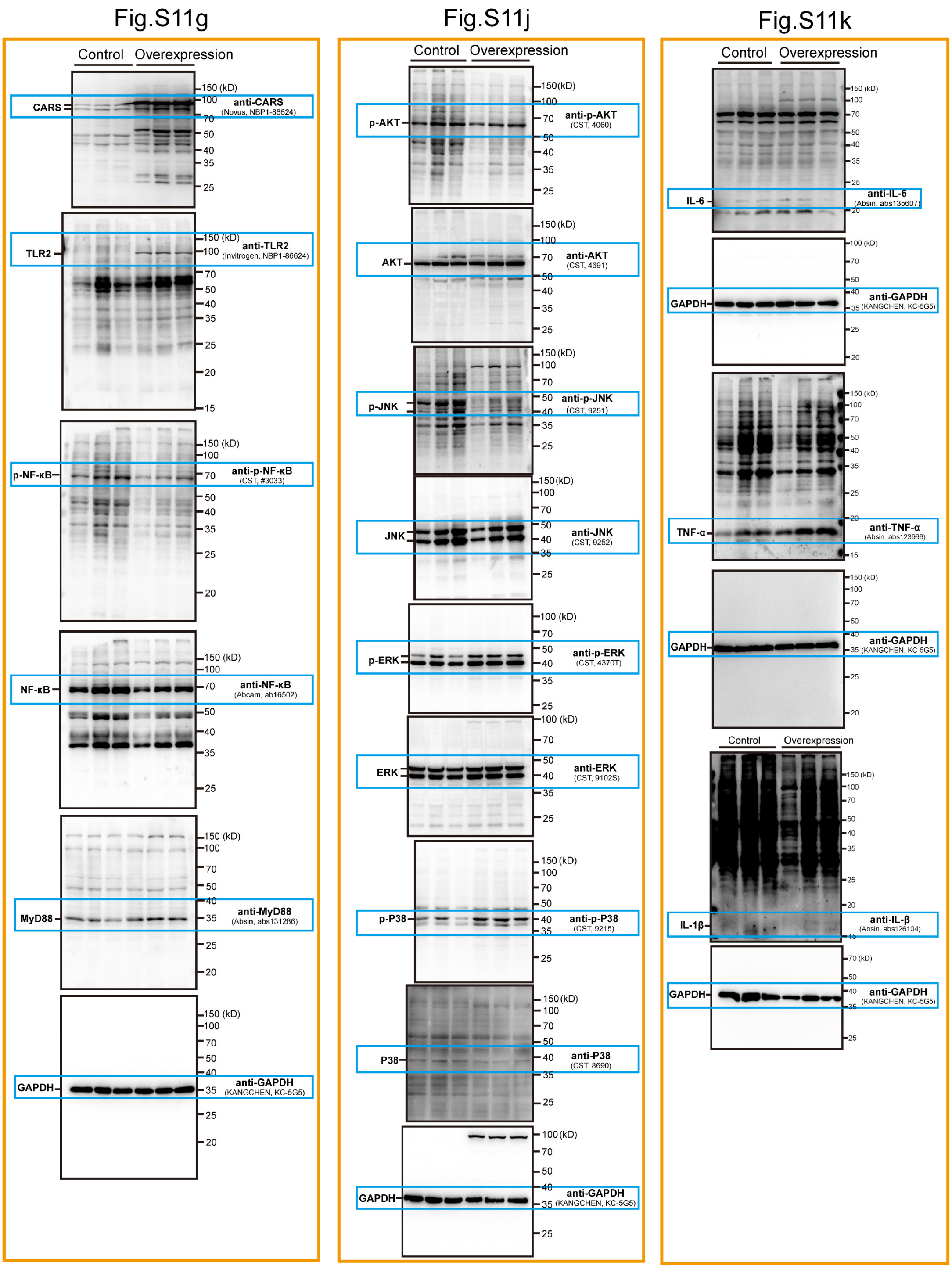

Fig. S12 The unedited western blotting gels for Fig. S11g, S11j andS11k.

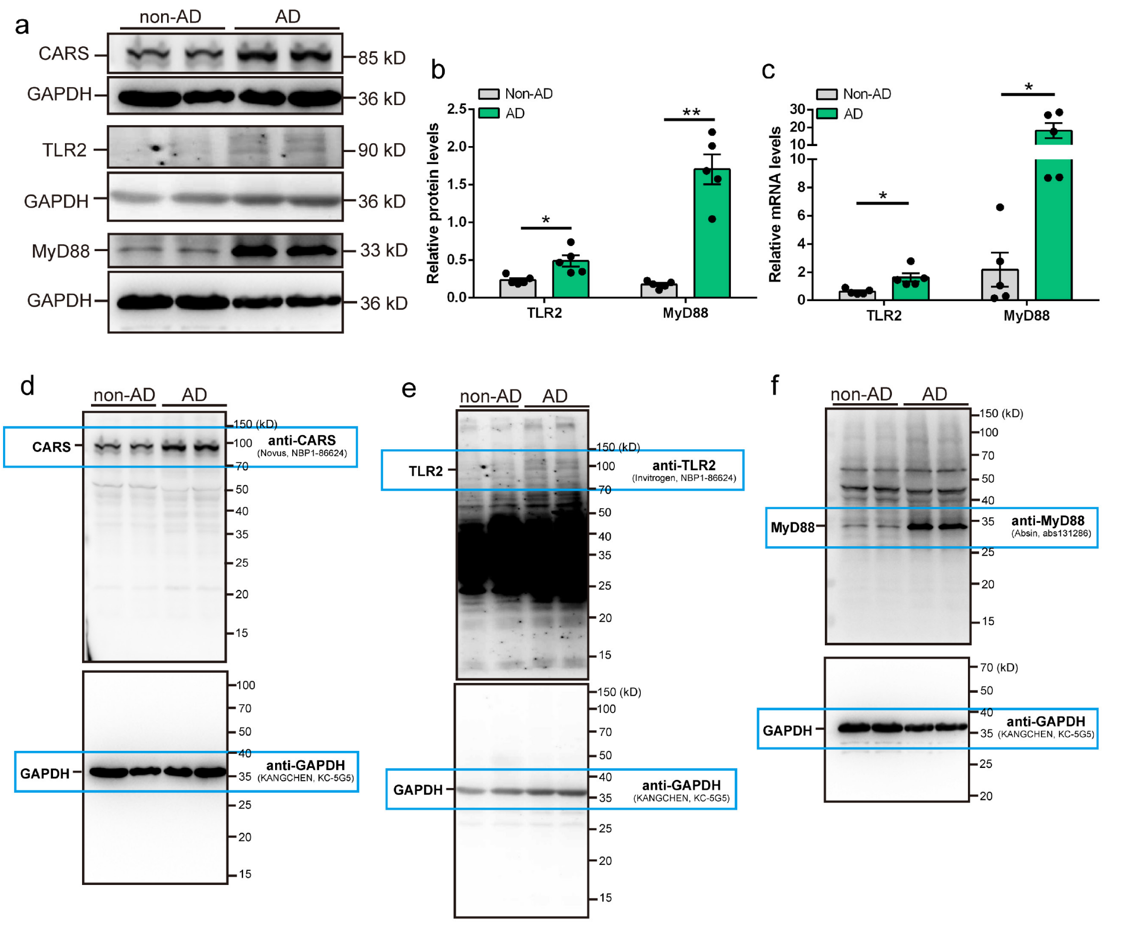

Fig. S13 Increased protein and mRNA levels of TLR2 and MyD88 in the temporal cortex of subjects with AD. a–c Representative western blotting images of CARS, TLR2 and MyD88 protein expression (**a**), and quantification of TLR2 and MyD88 protein expression (**b**) and mRNA levels (**c**) in the non-AD and AD human temporal cortex. **d–f** The unedited western blotting gels for **a**. Data are shown as mean ± SEM; *p <0.05, **p <0.01.

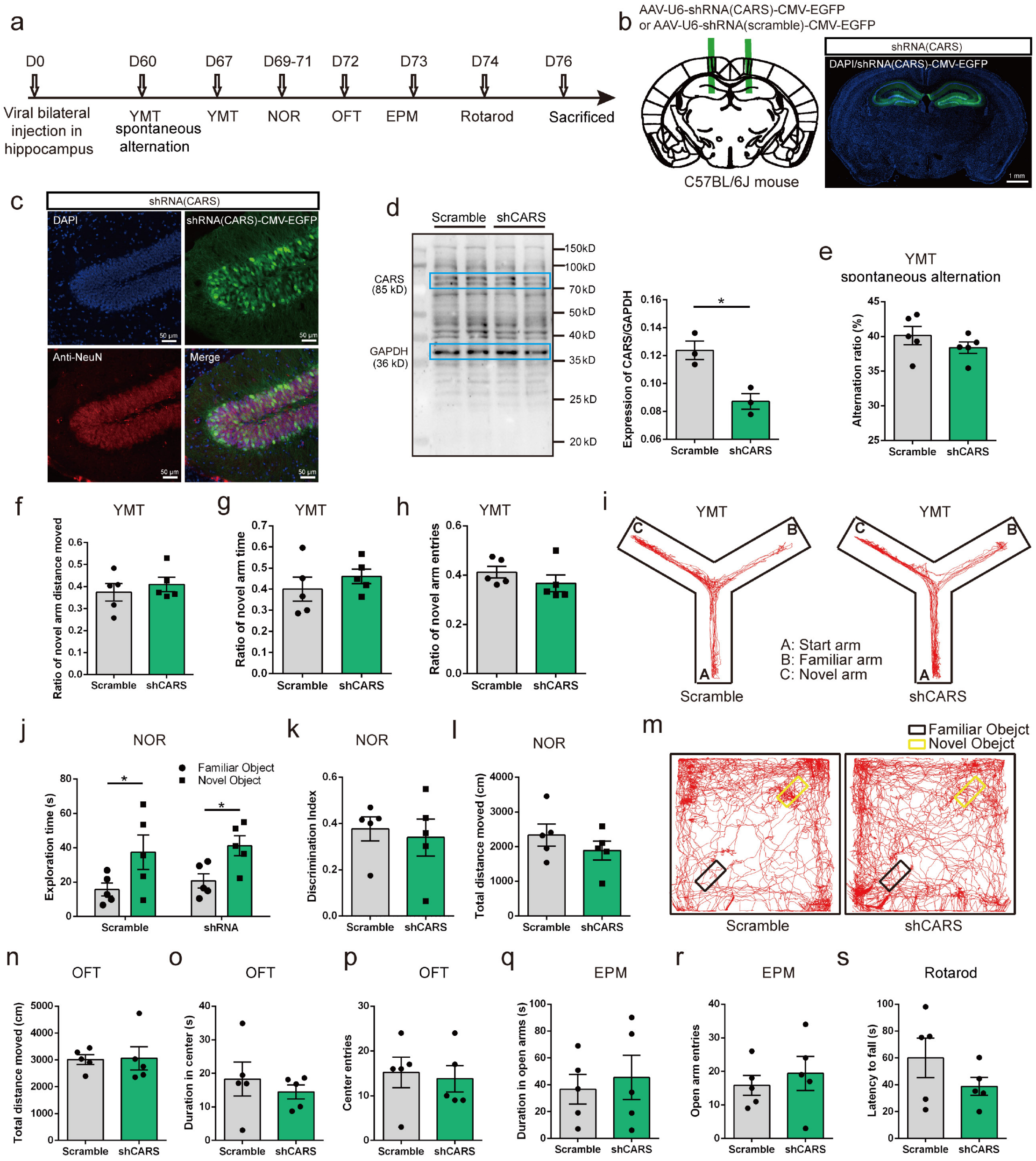

Fig. S14 Knockdown of CARS in hippocampal neurons dose not affect memory of C57BL/6J mice. **a** The schedule of viral injection and the following behavioral tests. **b** Left: bilateral injection of AAV-U6-shRNA(CARS)-CMV-EGFP (shCARS) or AAV-U6-shRNA(scramble)-CMV-EGFP (scramble) into the hippocampus of C57BL/6J mice. Right: a representative image of infected hippocampus; scale bar: 1 mm. **c** Immunostaining of NeuN in the hippocampus after the expression of shCARS; scale bars: 50 μm. **d** Left, western blotting of CARS protein expression in the hippocampus of scramble or shCARS mice. Right, quantification of CARS protein expression in

the hippocampus. **e** Spontaneous alternation behavior determined by Y-maze test (YMT). **f–h** Ratios of distance moved (**f**), time spent (**g**) and entries (**h**) in the novel arm in the YMT. **i** Representative activity traces of scramble and shCARS mice in the YMT. **j–l** The exploration time towards familiar and novel objects (**j**), discrimination index (**k**) and total distance moved (**l**) in the novel object recognition (NOR) test. **m** Representative activity traces of scramble and shCARS mice in the NOR test. **n–p** The total distance moved (**n**), time spent (**o**) and entries (**p**) in the center area in the open field test (OFT). **q** and **r** The time (**q**) and entries (**r**) in the open arms in the elevated plus maze (EPM) test. **s** The average latency to fall in the rotarod test. Data are shown as mean ± SEM; $*p < 0.05$.

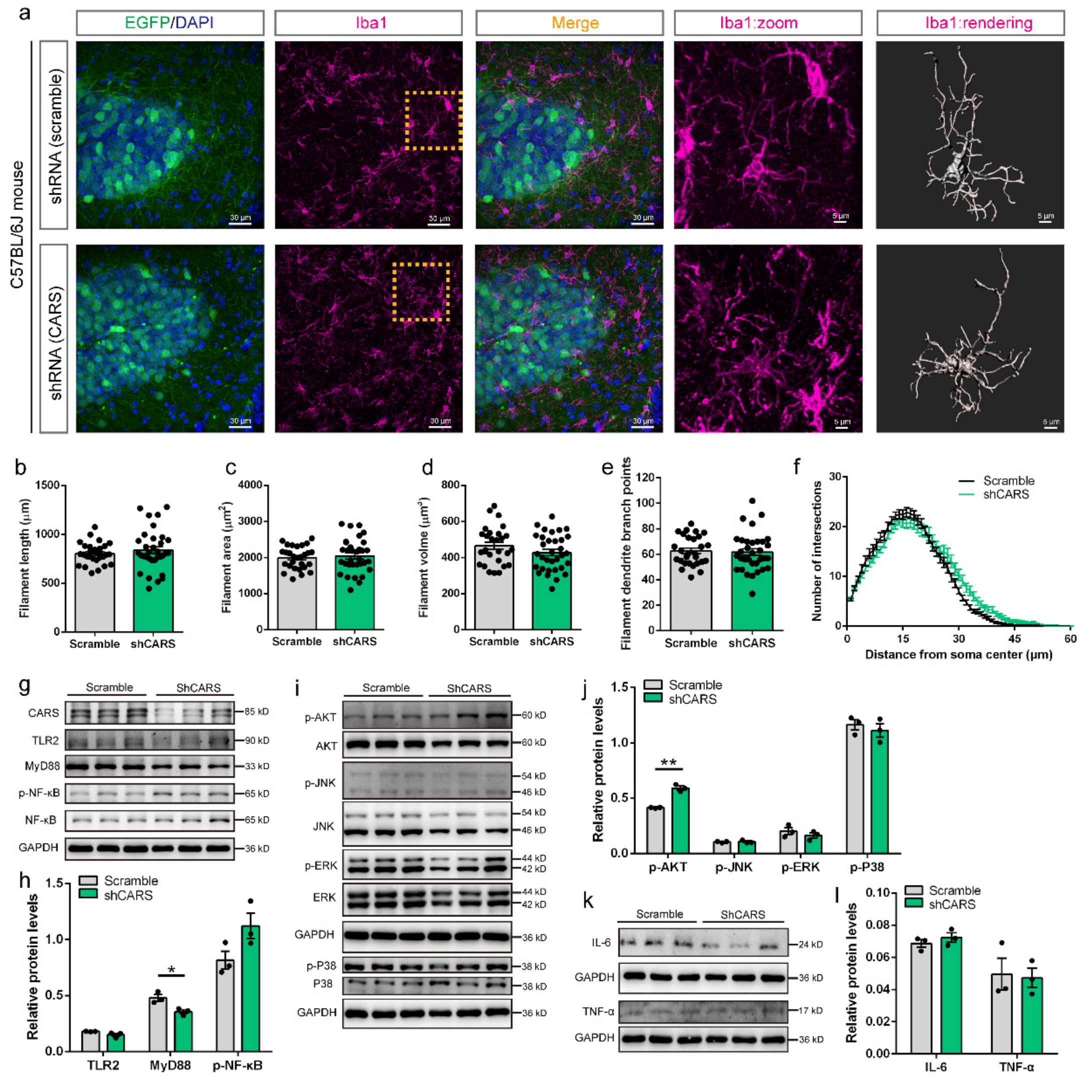

Fig. S15 Knockdown of CARS in hippocampal neurons induces downregulation of the TLR2/MyD88 pathway, while does not affect microglial morphology in hippocampus of C57BL/6J mice. **a** Representative images of Iba1 immunostaining (pink) and three-dimensional (3D) reconstruction (gray) of microglia in the hippocampus from C57BL/6J mice treated with AAV-U6-shRNA(CARS)-CMV-EGFP (shCARS) or AAV-U6-shRNA(scramble)-CMV-EGFP (scramble). Zoomed in view and rendering are shown in the right two panels respectively. Scale bars: 30 μm (overview) and 5 μm (zoomed in view and rendering). **b**–**e** Imaris-based automated quantification of Iba1^{+} microglial filament length (**b**), filament area (**c**), filament volume (**d**), and numbers of dendrite branch points (**e**) in the hippocampus from scramble and shCARS C57BL/6J mice. **f** Sholl

analysis of microglial morphology in scramble and shCARS C57BL/6J mice. **g** and **h** Representative western blotting images (**g**) and quantification (**h**) of TLR2, MyD88 and p-NF-κB protein expression in the hippocampus of scramble and shCARS mice. **i** and **j** Representative western blotting images (**i**) and quantification (**j**) of p-AKT, p-JNK, p-ERK and p-P38 protein expression in the hippocampus of scramble and shCARS mice. **k** and **l** Representative western blotting images (**k**) and quantification (**l**) of IL-6 and TNF-α protein expression in the hippocampus of scramble and shCARS mice. Data are shown as mean ± SEM; *p < 0.05, **p < 0.01.

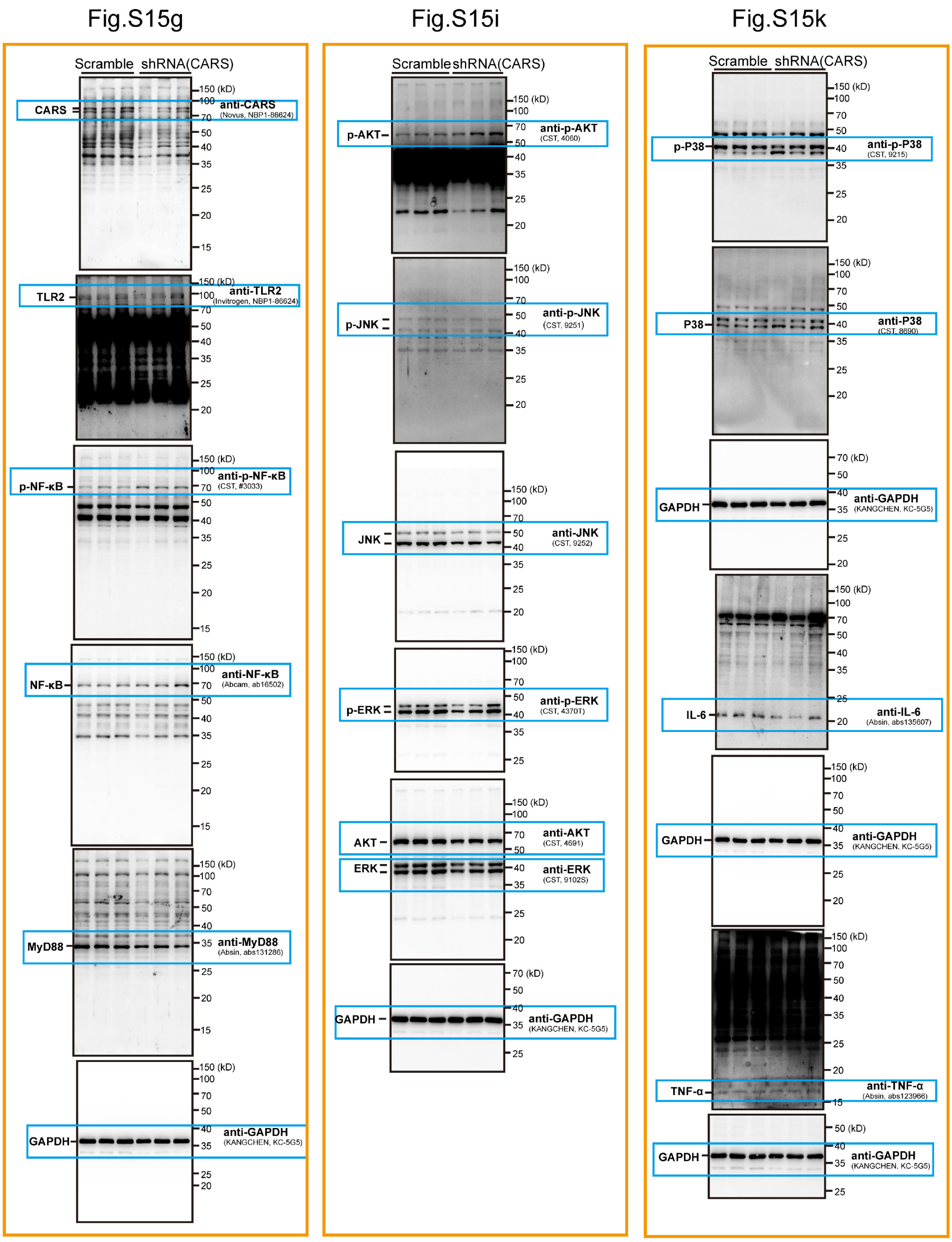

Fig. S16 The unedited western blotting gels for Fig. S15g, S15i and S15k.

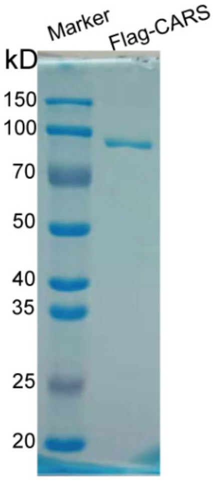

Fig. S17 Purified Flag-tagged CARS. Images showed the purified Flag-Tagged CARS on SDS-PAGE.

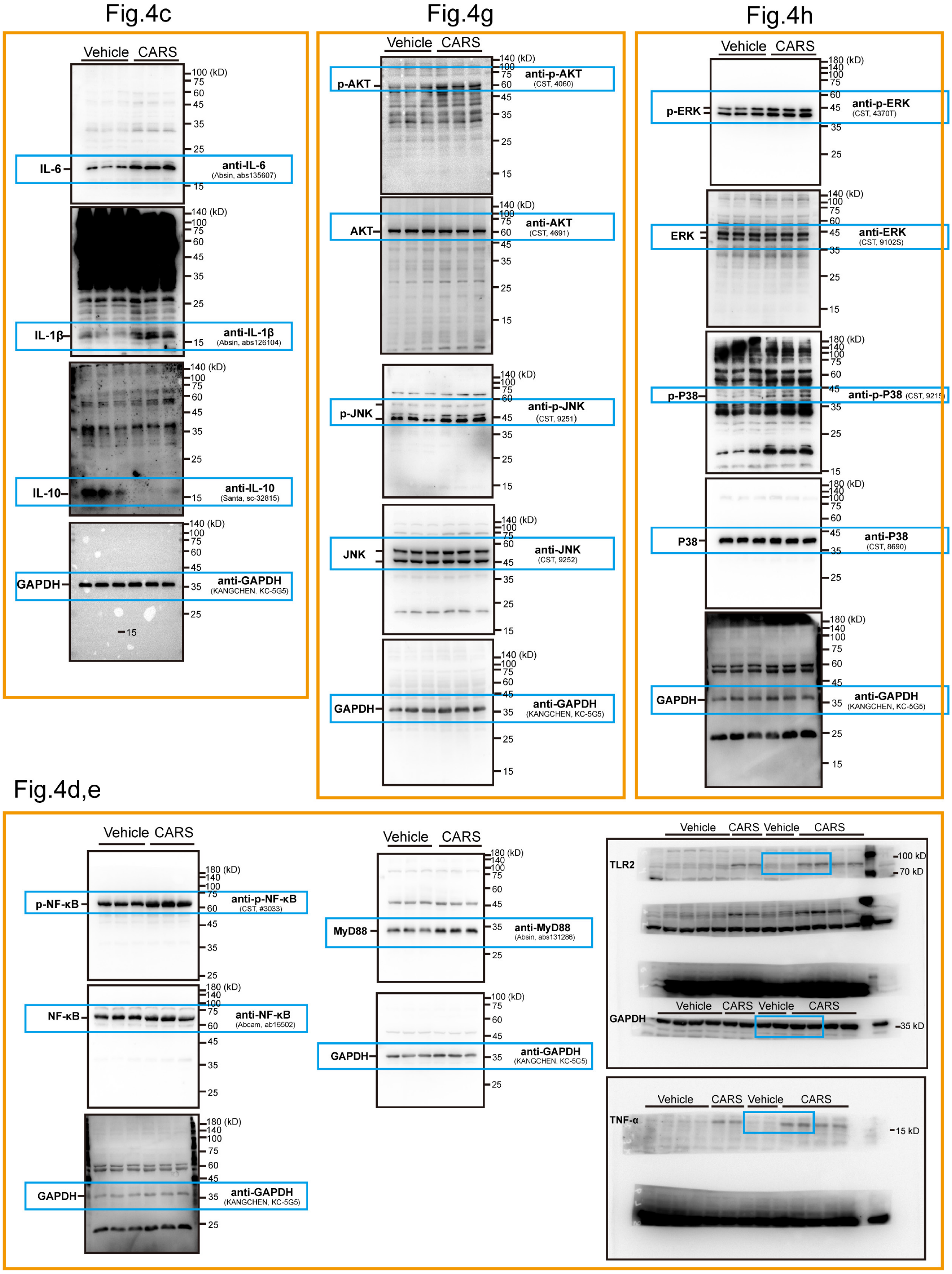

Fig. S18 The unedited western blotting gels for Fig. 4c, 4d, 4e, 4g and 4h.

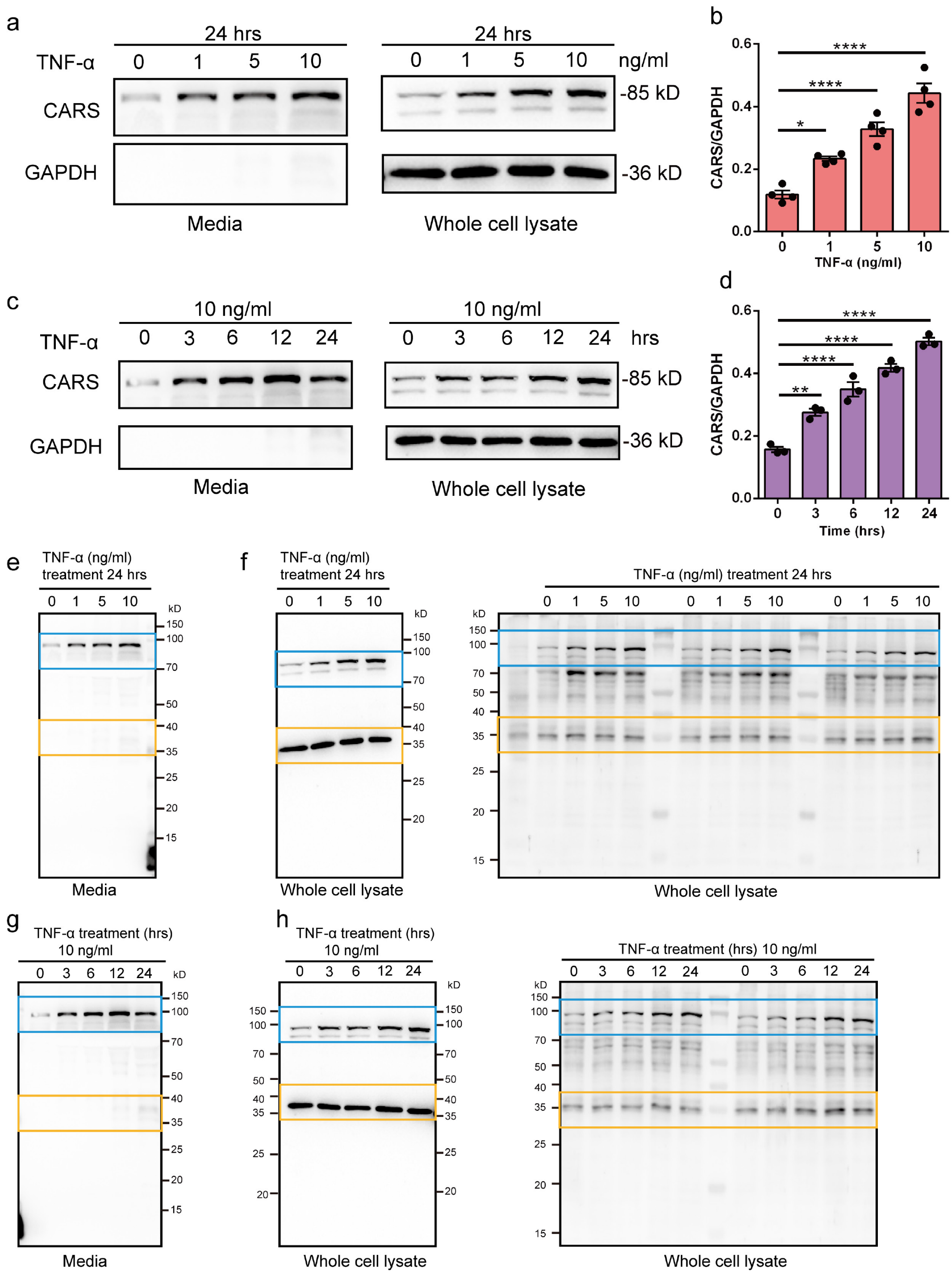

Fig. S19 TNF-α treatment results in increased levels of CARS protein expression. a and **b** Representative western blotting images (**a**) and quantification (**b**) of CARS protein levels in whole cell lysate of SHSY5Y cells treated with different dose of TNF-α (0, 1, 5 and 10 ng/ml) for 24 h. **c** and **d** Representative western blotting images (**c**) and quantification (**d**) of CARS protein levels in whole cell lysate of SHSY5Y cells treated with TNF-α (10 ng/ml) for different

time periods (0, 3, 6, 12 and 24 h). **e–h** The unedited western blotting gels for **a–d**. Data are shown as mean ± SEM; *p < 0.05, ** p < 0.01, *****p < 0.0001.

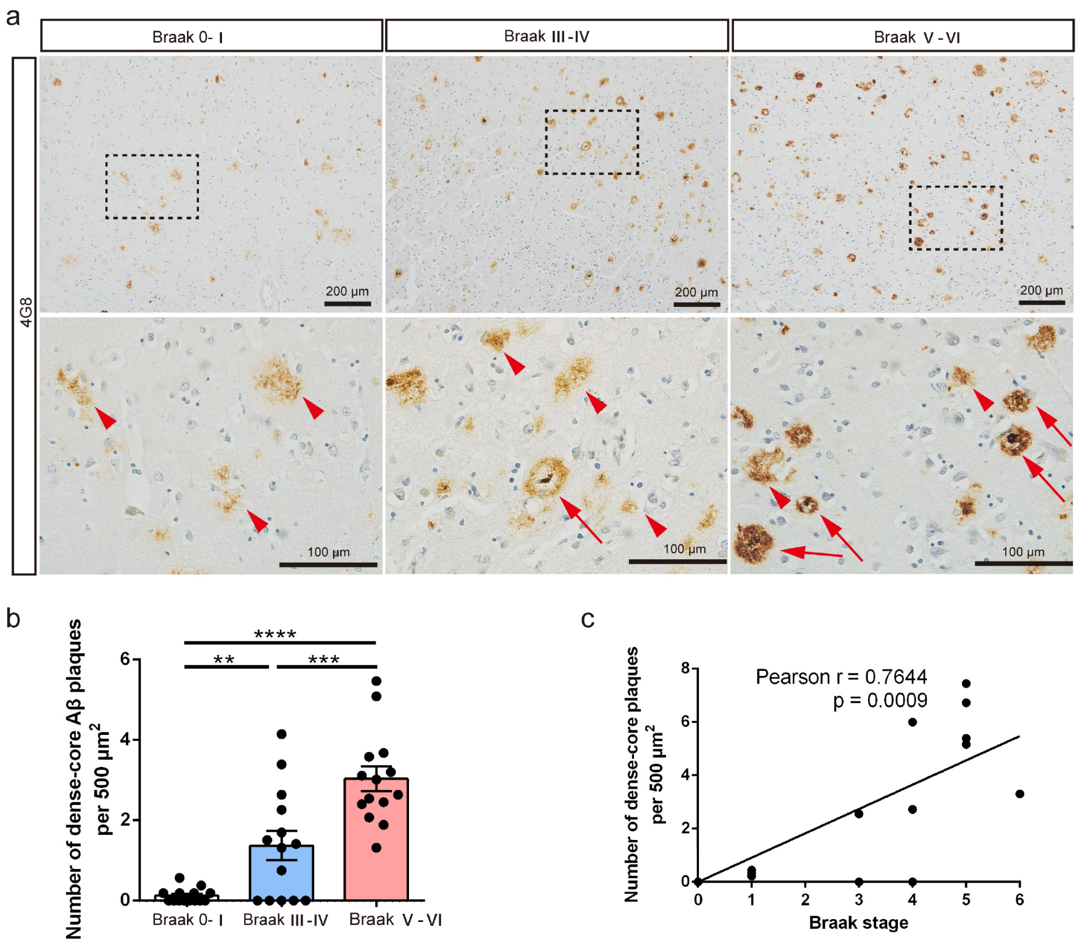

Fig. S20 Increased dense-core Aβ plaques in AD. a Representative images showing 4G8 (anti-β-amyloid antibody) labeled Aβ plaques in the temporal cortex of subjects with Braak stages. Magnifications of black dotted boxes in the upper panels are shown in the lower panels. Red arrowheads denote diffusive Aβ plaques, red arrows denote dense-core Aβ plaques. Scale bars: upper, 200 μm; lower, 100 μm. **b** Quantification of the density of dense-core plaques. **c** Correlation between the density of dense-core plaques in the temporal cortex and Braak stage of control and AD subjects. Data are shown as mean ± SEM; $^{**}p < 0.01$, $^{***}p < 0.001$, $^{****}p < 0.0001$.

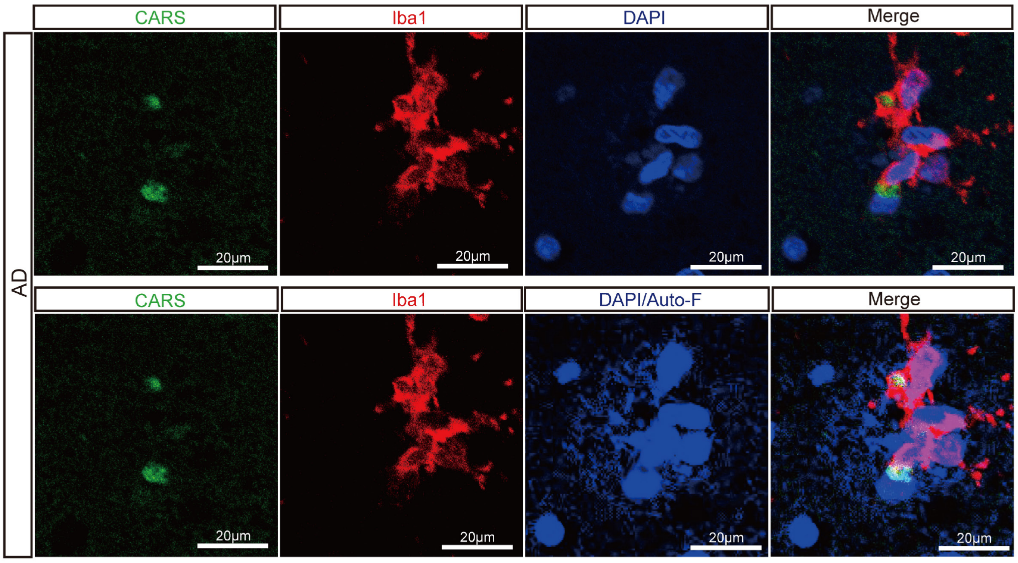

Fig. S21 CARS is accumulated within dense-core Aβ plaques along with recruitment of ameboid microglial cells in AD. Upper panels: Representative images of immunostaining for CARS (green), Iba1 (red), and DAPI (blue) in the AD human temporal cortex. Lower panels: Representative images of the autofluorescence (auto-F, blue) of dense-core Aβ plaque excited at 405 nm and immunostaining for CARS (green), Iba1 (red), and DAPI (blue) in the AD human temporal cortex. Scale bars: 20 μm.
